# Repeated mutation of a developmental enhancer contributed to human thermoregulatory evolution

**DOI:** 10.1073/pnas.2021722118

**Published:** 2021-04-13

**Authors:** Daniel Aldea, Yuji Atsuta, Blerina Kokalari, Stephen F. Schaffner, Rexxi D. Prasasya, Adam Aharoni, Heather L. Dingwall, Bailey Warder, Yana G. Kamberov

**Affiliations:** ^a^Department of Genetics, Perelman School of Medicine, Philadelphia, PA 19104;; ^b^Genetics Department, Harvard Medical School, Boston, MA 02115;; ^c^Broad Institute of Massachusetts Institute of Technology and Harvard, Cambridge, MA 02138;; ^d^Epigenetics Institute, Department of Cell and Developmental Biology, Perelman School of Medicine, Philadelphia, PA 19104

**Keywords:** human evolution, Engrailed 1, eccrine gland, regulatory evolution, sweat

## Abstract

Humans sweat to cool their bodies and have by far the highest eccrine sweat gland density among primates. Humans’ high eccrine gland density has long been recognized as a hallmark human evolutionary adaptation, but its genetic basis has been unknown. In humans, expression of the *Engrailed 1* (*EN1*) transcription factor correlates with the onset of eccrine gland formation. In mice, regulation of ectodermal *En1* expression is a major determinant of natural variation in eccrine gland density between strains, and increased *En1* expression promotes the specification of more eccrine glands. Here, we show that regulation of *EN1* has evolved specifically on the human lineage to promote eccrine gland formation. Using comparative genomics and validation of ectodermal enhancer activity in mice, we identified a human *EN1* skin enhancer, hECE18. We showed that multiple epistatically interacting derived substitutions in the human ECE18 enhancer increased its activity compared with nonhuman ape orthologs in cultured keratinocytes. Repression of hECE18 in human cultured keratinocytes specifically attenuated *EN1* expression, indicating this element positively regulates *EN1* in this context. In a humanized enhancer knock-in mouse, hECE18 increased developmental *En1* expression in the skin to induce the formation of more eccrine glands. Our study uncovers a genetic basis contributing to the evolution of one of the most singular human adaptations and implicates multiple interacting mutations in a single enhancer as a mechanism for human evolutionary change.

## Significance

One of the most distinctive physiological traits differentiating humans from other primates is a reliance on sweating to cool off. The effectiveness of human thermoregulatory sweating is underlain by the evolution of a dramatically increased density of water-secreting eccrine sweat glands in human skin relative to that of other primates. Here, we show that the accumulation of human-specific mutations in a developmental enhancer collectively promoted the production of eccrine glands in humans by up-regulating the expression of the *Engrailed 1* transcription factor in the skin. This study reveals a mechanism that contributed to the evolution of humans’ signature thermoregulatory capabilities and underscores the importance of regulatory evolution in generating the modern human form.

Because of their furlessness, humans are often called the “naked ape,” but humans can also be referred to as the “sweaty ape” (1). The evaporation of sweat is the major mechanism by which humans dissipate body heat (2), and humans have thermally induced sweat rates that are four to 10 times higher than those of chimpanzees, secreting 1 L or more of sweat per hour (3, 4). This dependence on sweating relies on two critical adaptations. The first is our loss of fur; because fur reduces airflow over the skin (5), human furlessness is thought to be an adaptation that enhances sweat evaporation (6–9). The second is the high density of eccrine sweat glands in human skin (4, 7, 8, 10). It is these glands that secrete the water humans vaporize for evaporative cooling (2), and it is their high density that make humans’ copious sweat production possible. Indeed, eccrine glands are the predominant appendages of human skin, with densities exceeding 200 glands/cm^2^ in regions such as the face (10). In comparison, mean eccrine gland densities of macaques and chimpanzees are 10 times lower (10). The importance of eccrine glands in humans is underscored by the risk of hyperthermia in individuals with reduced numbers of these organs (11). How and when humans diverged from other primates to evolve their dramatically elaborated eccrine gland density is unknown.

The evolution of humans’ high eccrine gland density required modification to the program controlling the number of eccrine glands specified during development. Much of our understanding of this developmental program comes from studies of eccrine gland formation in mice. Mice retain the ancestral condition of having eccrine glands only in the volar (palmar/plantar) skin of the paws, where eccrine gland secretions regulate frictional contact with underlying surfaces (12). Here, eccrine glands are found in elevations of the volar skin called footpads and in the intervening interfootpad space (IFP) of the mouse paw. Like hair follicles and mammary glands, eccrine glands are a type of ectodermal appendage and derive from the basal keratinocyte layer of the skin. Components of core developmental pathways including Wnt, Fgf, Eda, and Bmp are implicated in the induction and patterning of ectodermal appendages, including eccrine glands (13–17). In contrast, we and others have previously shown that the transcription factor Engrailed 1 (En1) plays a restricted role in specifically promoting the formation of eccrine glands in mice (13, 18, 19). *En1* is expressed throughout the basal keratinocyte layer of developing mouse volar skin and is specifically up-regulated in eccrine gland placodes of the IFP (18). *En1* knockout mice fail to form eccrine glands, and decreasing *En1* levels results in the specification of fewer eccrine glands in mouse volar skin (18–20). In fact, intrinsic differences in the *cis* regulation of *En1* are a primary cause of the fourfold greater abundance of eccrine glands in the IFP of FVB/N compared with C57BL/6N mouse strains (18). Strikingly, *EN1* is up-regulated in human fetal ectoderm coincident with the onset of eccrine gland specification (13). In light of these findings, we investigated whether evolutionary changes leading to increased ectodermal *EN1* expression during development could be an underlying mechanism for the adaptive increase in human eccrine gland density.

## Results

### Identification of Candidate *En1* Enhancers in the Skin.

There are no human-specific variants that distinguish the human *EN1* coding sequence from that of other Catarrhines, the group of primates which includes humans, apes, and Old World monkeys (monkeys of Asia and Africa, OWMs), and most evolutionarily relevant changes are likely to reside in regulatory DNA (21). We therefore first investigated whether humans have altered *EN1* regulation by screening for enhancers with the potential to control this locus during eccrine gland development.

**Fig. 1.**
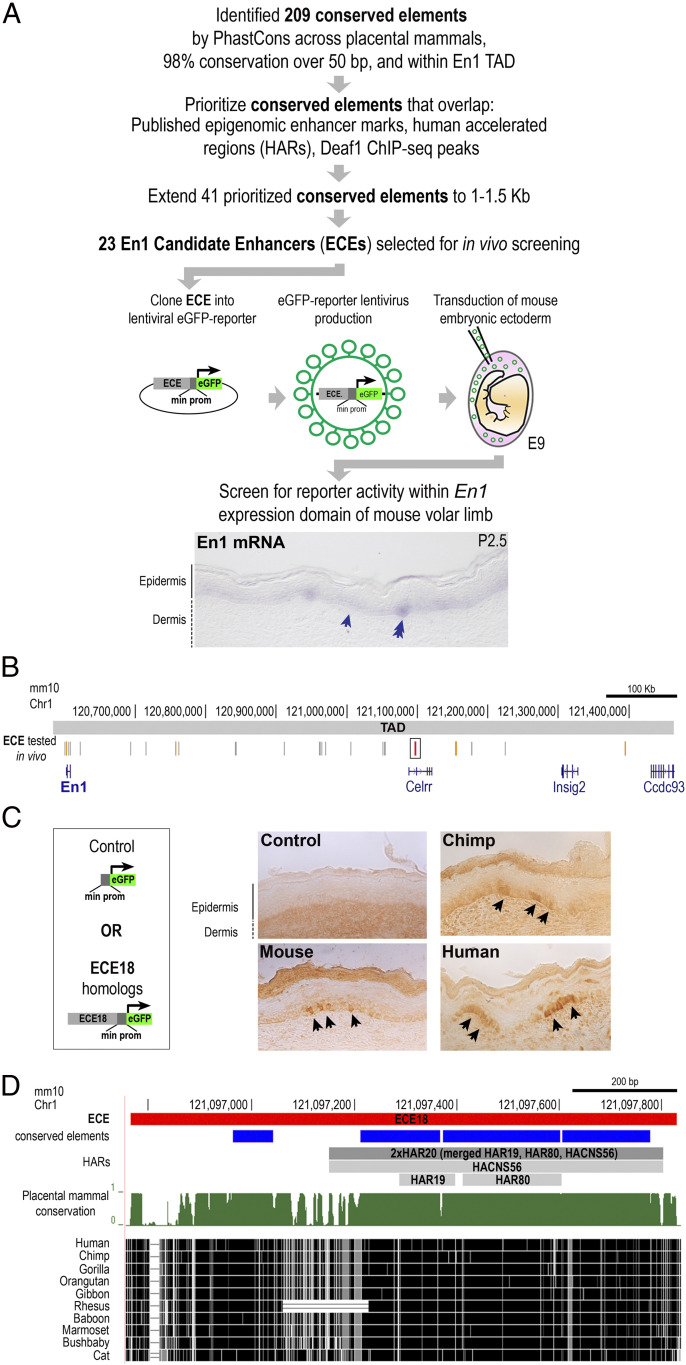
Identification of *Engrailed 1* candidate enhancer ECE18. (*A*) Strategy to identify putative developmental *En1* enhancers. Staining for *En1* mRNA (purple) in mouse volar limb skin at P2.5 is shown The basal keratinocyte layer (arrow) and eccrine placode (double arrow) are shown. (*B*) The location in mm10 of ECEs tested in vivo (vertical gray lines). ECE18 is in red and boxed and other positive ECEs are highlighted in orange. (*C*) Representative images of mouse, chimpanzee, and hECE18 transgenic P2.5 volar limb stained with anti-GFP antibody. eGFP (black arrow) is visualized using HRP-DAB coupled immunohistochemistry. (*D*) Sequence alignment and evolutionarily features of ECE18, which contains four conserved elements by phastCons (blue rectangles) and overlaps the human accelerated regions HACNS56, HAR19, and HAR80 (collectively called 2xHAR20).

Direct interrogation of human developmental enhancers in this context is confounded by the fact that there are no in vitro systems that recapitulate human eccrine gland development or indeed the development of these appendages from any other species. Moreover, eccrine gland anlagen, or placodes, begin to form during the second trimester in humans (22). Accordingly, we used a candidate-based screen to identify putative enhancers. In general, patterns of ectodermal *EN1* expression are similar across mammals in body regions where eccrine glands form (13, 18, 23), and therefore we sought to identify putative *EN1* enhancers based on evidence of evolutionary sequence conservation (Fig. 1*A*). We restricted our screen to noncoding DNA within the genomic interval spanned by the *EN1* topologically associated domain (TAD) as defined by Hi-C in normal human cultured keratinocytes (Chr2: 118000000–118880000 [hg38]) since studies on the compartmentalization of mammalian genomes suggest that elements controlling the expression of *EN1* are likely to be located within this interval (24, 25). Importantly, the genomic interval spanned by the human *EN1* TAD is in a region of conserved synteny to the mapped mouse *En1* TAD (Chr1: 120583423–121463423 [mm10]) (24). Using phastCons (26), we called 209 evolutionarily conserved elements within the *EN1* TAD that were at least 50 base pairs (bps) using an empirically determined threshold of ≥98% probability of being conserved in placental mammals (*SI Appendix*, Fig. S1*A*). We prioritized elements based on overlap with published datasets of genomic regions containing epigenomic marks suggestive of enhancer presence from both mouse (27, 28) and human datasets and also with published catalogs of human genomic regions showing evidence of accelerated evolution (29–31). Specifically, we prioritized elements overlapping deoxyribonuclease (DNase) I hypersensitivity, *En1* Capture-C, and H3K27Ac chromatin immunoprecipitation sequencing (ChIP-seq) peaks from embryonic mouse limbs in which *En1* is expressed throughout the ventral limb ectoderm (27, 28). Elements were also prioritized if they overlapped ChIP-seq peaks for DEAF1 in human keratinocytes since we previously reported that this transcription factor positively regulates *Enrgailed 1* expression in both human and mouse skin cells (20). In addition, we prioritized any elements that overlapped with annotated human accelerated regions (HARs), which are a class of genomic elements that are highly conserved across vertebrates but are exceptionally diverged in humans, suggestive of evolutionary importance in our species (29–31). This prioritization scheme generated a list of 41 priority conserved elements. Using each priority element as a kernel, we expanded these genomic regions to 1,000 to 1,500 bps to accommodate typical enhancer lengths, which tend to be hundreds of base pairs long; combining overlapping regions, this collapsed our list to 23 *Engrailed 1* candidate enhancers (ECEs) for functional testing (Fig. 1 *A* and *B* and *SI Appendix*, Fig. S1*B*).

To determine if the ECEs could function as enhancers in *En1*-expressing keratinocytes during eccrine gland development, we tested whether each of the 23 ECEs could activate reporter expression in the basal keratinocyte layer of mouse volar skin on postnatal day (P) 2.5, which is the stage when eccrine gland placodes are being specified in this region (18). To this end, we used lentiviruses encoding the mouse ortholog of each ECE upstream of a minimal promoter and an eGFP reporter cassette to stably transduce embryonic mouse ectoderm and generate skin-specific transgenic ECE reporter mice (Fig. 1*A*) (32–34).

Of the 23 tested ECEs, five consistently produced multiple eGFP-positive keratinocyte clones (clusters of keratinocytes that are derived from the same ancestral progenitor) in the basal *En1*-positive layer of mouse volar skin on P 2.5 (Fig. 1 *B* and *C* and *SI Appendix*, Fig. S1*B*). At this stage, *En1* is critical for specifying the number of eccrine glands in the IFP and is expressed throughout basal keratinocytes of the distal volar skin and focally up-regulated in IFP eccrine placodes (Fig. 1*A*) (18). The five positive ECEs consisted of a fragment of the *En1* promoter (ECE2, Chr1: 120601976–120602486 [mm10]) and four elements located downstream of *En1* (ECE8, Chr1: 120756823–120757766; ECE18, Chr1: 121096764–121097826; ECE20, Chr1: 121176848–121178300; and ECE23, Chr1: 121394405–121395702 [mm10]) (Fig. 1*C* and *SI Appendix*, Figs. S1*B* and S2*A*). All positive ECEs also produced positive clones within the basal ectoderm of the volar footpads and differentiating eccrine glands therein, indicating these ECEs can function at later stages of eccrine gland development (*SI Appendix*, Fig. S2*B*). We noted that ECE2, ECE18, and ECE20 lentivirus-mediated transgenic mice also had eGFP-positive clones in the dorsal limb skin, which is outside of the *En1*-positive domain in mice (*SI Appendix*, Fig. S2*C*). This observation is intriguing given that *EN1* expression in the basal ectoderm is expanded to the nonvolar skin in humans.

### Enhanced Activity of the Human ECE18 Ortholog.

Among the five positive ECEs identified in our in vivo screen, we focused our subsequent analyses on ECE18, which overlapped the most epigenomic signatures that are suggestive of enhancer activity from published datasets (*SI Appendix*, Fig. S1*A*). Moreover, since our goal was to identify ECEs with the potential to explain a human-specific phenotype, ECE18 additionally stood out as the element that contains the highest number of derived human mutations among the five positive ECEs and overlaps the HACNS56, HAR19, and HAR80 human accelerated regions (collectively named 2xHAR20) (29–31) (Fig. 1*D* and *SI Appendix*, Figs. S1*C* and S3*A*).

**Fig. 2.**
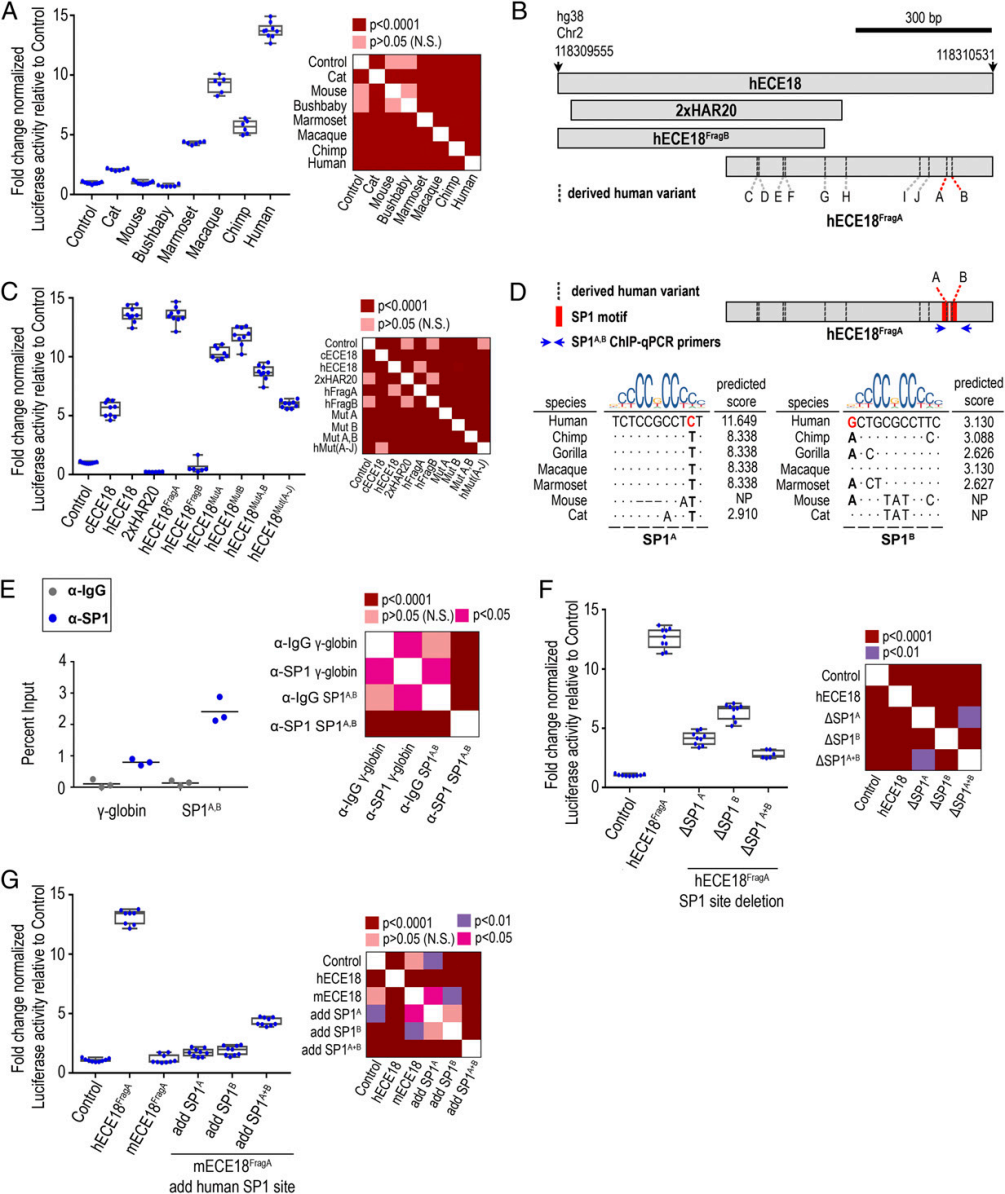
Repeated mutation of ECE18 produced human-specific gains in enhancer activity. (*A*) Fold induction of luciferase reporter activity relative to empty vector (Control) by ECE18 orthologs in cultured human keratinocytes. (*B*) Schematic of ECE18 variants tested in *C*. Derived human bases at A and B are highlighted in red, and the positions of the remaining derived human-specific base substitutions are shown as dashed vertical lines. (*C*) Localization of hECE18 enhancer activity. The fold change normalized luciferase activities of full-length hECE18, chimpanzee ECE18 (cECE18), human 2xHAR20, hECE18 fragment (Frag) A and B, and mutant hECE18 in which the indicated derived base is replaced by the ancestral ape base are plotted. Mutated base (Mut). All 10 derived human substitutions are mutated to ancestral ape bases in hECE18^MutA−J^. (*D*) Species alignment and predicted binding affinities for SP1 at SP1^A^ and SP1^B^. Derived human bases at A and B are in red. Dots indicate identity to human base. Blue arrows indicate the location of ChIP-qPCR primers used in *E*. (*E*) Enrichment of SP1 by ChIP-qPCR at hECE18 interval containing SP1^A^ and SP1^B^ motifs (SP1^A,^ ^B^) in human keratinocytes. Human γ-globin (HBG2) promoter is used as a negative control, and IgG or SP1 enrichment over the input for each set of primers is shown. Mean enrichment is reported across three independent experiments (line). (*F*) Fold induction of luciferase reporter by hECE18^FragA^ upon deletion of SP1^A^ and SP1^B^. (*G*) Fold induction of luciferase reporter by mouse ECE18^FragA^ (mECE18^FragA^) in human keratinocytes upon knock-in of human SP1^A^ and SP1^B^ motifs. Normalized Firefly luciferase activity is plotted as the fold change relative to Control (empty reporter vector). Firefly luciferase values are normalized to Renilla luminescence. The dots represent an individual biological replicate. Median (line), box (bounds 25 to 75%) and whiskers (min and max) plotted. Significance by one-way ANOVA. Tukey-adjusted *P* values are reported in heatmaps. Assays are performed in human GMA24F1A keratinocytes.

Consistent with having ectodermal enhancer capabilities during development, the human and chimpanzee orthologs of ECE18 (hECE18 and cECE18, respectively) induced eGFP-positive clones in limb skin in a pattern identical to that of mouse ECE18 (Fig. 1*C* and *SI Appendix*, Fig. S2 *B* and *C*). The mouse lentiviral transgenic system allows for comparisons of the spatial and temporal activities of candidate enhancers in the context of eccrine gland development. This assay is by nature qualitative since we cannot control for differences in transduction efficiency and in lentiviral expression due to integration site effects. Intriguingly, while all tested ECE18 orthologs behaved similarly in the mouse transgenic assay, the comparison of the activity of ECE18 orthologs in quantitative luciferase reporter assays in cultured human keratinocytes revealed dramatic differences in the potency of this enhancer between species (Fig. 2*A* and *SI Appendix*, Fig. S3 *B* and *C*). When cloned upstream of a minimal promoter driving luciferase expression, we found that anthropoid ECE18 orthologs produced the greatest-fold luciferase induction, while orthologs from species outside this primate infraorder induced little to no luciferase activity (Fig. 2*A*). Among tested orthologs, hECE18 was the most potent enhancer, producing on average a 13-fold increase in luciferase over control. Within Catarrhines, the group consisting of OWMs and apes, hECE18 activity was 2.6-fold and 1.4-fold higher relative to chimpanzee and macaque orthologs, respectively (Fig. 2*A*). These findings suggest gains in ECE18 potency specific to the anthropoid lineage and are consistent with further human-specific evolutionary changes to the functionality of this enhancer.

### Multiple Mutations Underlie Increased ECE18 Activity during Human Evolution.

Because ECE18 activity is higher in humans, we reasoned that human-specific sequences might underlie the potency of this enhancer in our species. To investigate the specific changes driving the higher activity of the human enhancer ortholog, we first tested the 2xHAR20 genomic fragment that contains the highest number of derived human substitutions in hECE18 for enhancer activity (Fig. 1*D* and *SI Appendix*, Fig. S3*A*). Human 2xHAR20 did not induce reporter expression in cultured human keratinocytes or in mouse transgenic assays, indicating that this region is not sufficient for enhancer activity (Fig. 2 *B* and *C*). We then split hECE18 into two partially overlapping fragments: hECE18^FragB^ (hg38 Chr2: 118309555–118310154 bp) and hECE18^FragA^ (hg38 Chr2: 118309932–118310531 bp) (Fig. 2*B* and *SI Appendix*, Fig. S3 *A* and *C*). hECE18^FragB^ did not induce reporter expression in vivo or in vitro (Fig. 2*C*). In contrast, hECE18^FragA^ recapitulated both the quantitative activity and spatial/temporal activity of full-length hECE18 in cultured human keratinocytes and P2.5 mouse transgenic skin, respectively (Fig. 2*C* and *SI Appendix*, Fig. S3 *C*–*E*). hECE18^FragA^ is highly conserved among modern humans, and we found that only a handful of low frequency variants detectably altered enhancer activity in vitro (*SI Appendix*, Fig. S3 *F* and *G*). ECE18^FragA^ orthologs from nonhuman anthropoids also recapitulated the quantitative activity of the respective full-length elements (*SI Appendix*, Fig. S3*E*). As with full-length hECE18, hECE18^FragA^ produced the same fold change increase in luciferase relative to tested nonhuman primate orthologs, consistent with the idea that this region is sufficient to explain enhancer activity in all species (Fig. 2*C* and *SI Appendix*, Fig. S3*E*).

We found that replacing even a portion of the genomic interval spanned by hECE18^FragA^ in hECE18 with the orthologous chimpanzee sequence was sufficient to reduce the activity of the enhancer in luciferase assays, indicating the importance of human sequence changes (*SI Appendix*, Fig. S3*H*). hECE18^FragA^ contains 12 derived human mutations relative to other apes, including two single nucleotide insertions and 10 single nucleotide substitutions (called here sites A to J) (*SI Appendix*, Fig. S3*I*). Deletion of the two derived insertions or individual mutation of substitutions C to J to the ancestral ape base in hECE18 produced little or no effect on enhancer activity (*SI Appendix*, Fig. S3 *J* and *K*). However, mutation of the derived human nucleotides at A and B, alone or in combination, to the ancestral ape bases resulted in substantial and significant reductions of enhancer activity, though not to chimp-like levels (Fig. 2*C*). Intriguingly, mutation of all 10 derived human substitutions (A to J) together to the ancestral ape bases reduced hECE18 activity to that of nonhuman apes (Fig. 2*C*). It is worth noting that five of the derived human-specific substitutions (namely C to G) that contribute to this increased potency are all located within the region overlapping 2xHAR20 (Fig. 2*B*). This indicates that while the accelerated region is not sufficient for enhancer activity, its evolution played a role in driving the full magnitude of increase in hECE18 enhancer function. Moreover, our data suggest that the epistatic effects of multiple human-specific variants located within the interval spanned by hECE18^FragA^ are responsible for the increased potency of this enhancer in humans relative to other apes. From an evolutionary perspective, this means that ECE18 was repeatedly mutated over the course of human evolution, which collectively produced the human enhancer phenotype.

### A Conserved Mechanism for ECE18 Evolution.

In silico motif analysis revealed that sites A and B lie within tandem predicted binding motifs for the transcripton factor SP1, which we named SP1^A^ and SP1^B^, respectively (Fig. 2*D*). We confirmed SP1 occupancy by ChIP-qPCR in the region spanned by SP1^A^ and SP1^B^ (Fig. 2*E*). Deleting SP1^A^ or SP1^B^ in hECE18^FragA^ reduced luciferase levels to less than half that of the wild-type human enhancer, with loss of both motifs reducing enhancer activity nearly to control levels (Fig. 2*F*).

Overall, in silico analysis predicted anthropoids to have the highest affinity SP1^A^ and SP1^B^ motifs among mammals, and ECE18 anthropoid orthologs did prove to have the highest activity in vitro (Fig. 2 *A* and *D*). The correlation between higher affinity motifs at SP1^A^ and SP1^B^ and increased ECE18 potency was also observed within anthropoids, with humans having the highest affinity sites among apes and the quantitatively strongest enhancer in keratinocytes (Fig. 2*A*). This may also explain our observation that OWM enhancer orthologs were more active in luciferase assays than those of nonhuman apes, since OWMs have an identical SP1^B^ motif to humans (Fig. 2*A* and *SI Appendix*, Fig. S3 *E* and *L*). Consistent with a model in which sequence variation at SP1^A/B^ underlies phylogenetic variation in ECE18 potency, the introduction of human SP1^A^ and SP1^B^ motifs into mouse ECE18^FragA^, which is not predicted to bind SP1 at either position, additively increased luciferase induction by the mouse enhancer (Fig. 2*G*). Collectively, these data point to a common mechanism of variation at SP1 binding motifs as a conserved means to modulate ECE18 enhancer potency not only across mammals but also specifically within anthropoids.

### ECE18 Modulates Endogenous *EN1* Expression in Human Keratinocytes.

**Fig. 3.**
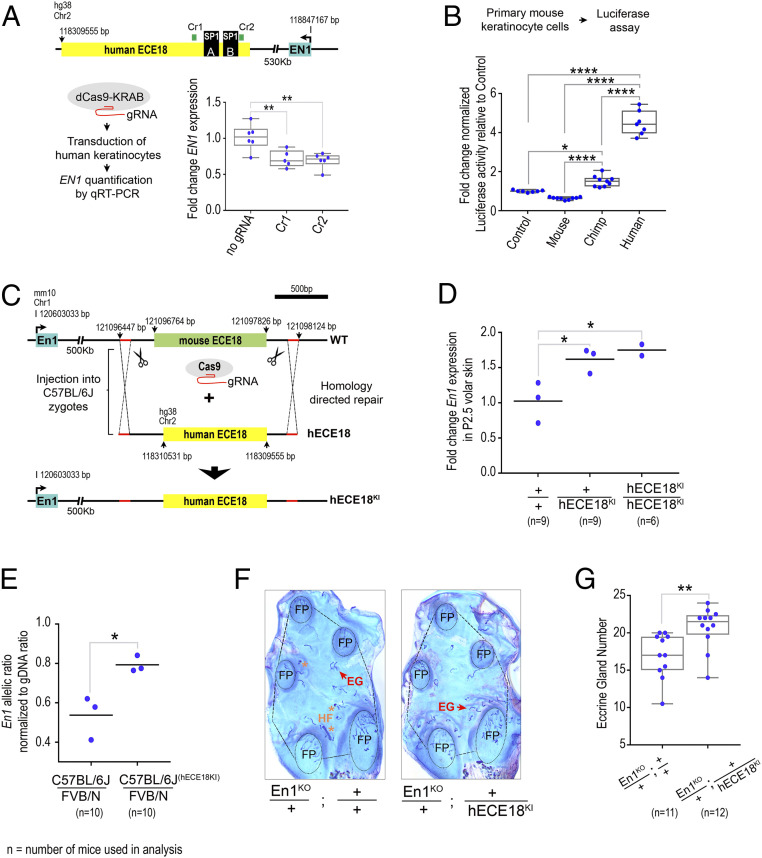
hECE18 positively regulates *Engrailed 1* to promote eccrine gland formation. (*A*) Fold change normalized *EN1* mRNA by qRT-PCR in human GMF24F1A cultured keratinocytes upon dCAS9-KRAB–mediated hECE18 repression. The schematic of strategy and relative positions of *EN1*, SP1^A^ and SP1^B^, and Cr1 and Cr2 gRNA targets is shown. The fold change calculated relative to dCAS9-KRAB transduction alone is shown. (*B*) Fold luciferase induction relative to empty reporter vector (Control) by ECE18 orthologs in primary mouse keratinocytes. (*C*) Strategy to generate hECE18 knock-in (hECE18^KI^) mouse model. (*D*) Fold change in *En1* mRNA by qRT-PCR in P2.5 volar forelimb skin of wild-type (^+/+^), hECE18^KI^ heterozygote (hECE18^KI^/+), and hECE18^KI^ homozygote (hECE18^KI^/hECE18^KI^) mice relative to wild type. (*E*) Normalized ratio of C57BL/6J:FVB/N *En1* allelic expression in volar forelimb skin of wild-type (C57BL/6J/FVB/N) and hECE18^KI^ (C57BL/6J^(hECE18KI)^/FVB/N) hybrid mice. The ratios were normalized to genomic DNA allelic ratio. (*F*) Representative stained epidermal preparations of volar forelimb skin from En1^KO^/+;^+/+^ and En1^KO^/+;+/hECE18^KI^ adult mice. The number of eccrine glands in the IFP area (outlined) and excluding the footpads (FP, circled) were quantified in analyses in *G*. Hair follicle (HF, *), eccrine gland (EG). (*G*) Quantification of IFP eccrine glands in En1 ^KO^/+;^+/+^ and En1 ^KO^/+;+/hECE18^KI^ mice. Each point represents the average number of eccrine glands in the IFP across both forelimbs of a mouse. (*A*, *B*, *G*) Median (line), 25 to 75% percentiles (box bounds) and min and max (whiskers) are plotted. (*D* and *E*) Genotype mean is shown as a line and each point represents a single biological sample of pooled volar skin from both forelimbs of at least three mice. (*A*, *B*, *D*) significance assessed by one-way ANOVA and Tukey-adjusted *P* values are reported. (*E* and *G*) Significance assessed by a two-tailed *t* test. ****P < 0.0001, **P < 0.01, *P < 0.05. (+) wild- type allele. (KO) knockout. (KI) knock-in. The dots represent an individual biological replicate.

Having identified an enhancer, hECE18, in which human-specific sequence evolution increased activity in keratinocytes, we investigated whether hECE18 regulates *EN1* in this context. We repressed the endogenous hECE18 enhancer in cultured human keratinocytes using a dCas9-Krüppel associated box (KRAB) domain fusion and guide RNAs (gRNAs) targeting the ECE18 genomic interval (Fig. 3*A*). We found that targeting the repressor complex to hECE18 reduced *EN1* messenger RNA (mRNA) by 40% on average relative to control (Fig. 3*A*). In contrast, repression of hECE18 did not decrease the expression of *Insig2* and *Ccdc93*, the other protein-coding gene loci located within the *EN1* TAD (*SI Appendix*, Fig. S4*A*). These data demonstrate that hECE18 acts to specifically up-regulate *EN1* expression in human keratinocytes (Fig. 3*A*).

### Human ECE18 Up-Regulates *En1* to Promote Eccrine Gland Specification.

To determine if hECE18 can function as a developmental enhancer of ectodermal *En1*, we turned to the mouse, which is an in vivo model system amenable to targeted genetic manipulation. We first determined if the higher potency of hECE18 could be recapitulated in this model. Consistently, hECE18 produced the greatest-fold increase in luciferase in cultured mouse primary keratinocytes (Fig. 3*B*). We noted, however, the relative increase over control was four-fold and 1.5-fold on average by hECE18 and cECE18, respectively, which was markedly lower than that observed in human skin cells for both orthologs. This suggests that the relative activity of ECE18 may be attenuated in this system. As in human keratinocytes, mouse ECE18 did not induce luciferase expression significantly. Moreover, in contrast to our findings in repressing the endogenous hECE18 enhancer in human keratinocytes, deletion of the homologous ECE18 sequence from the mouse genome did not alter ectodermal *En1* levels or eccrine gland number in mouse volar skin (*SI Appendix*, Fig. S5). These data show that though ECE18 is not essential for *En1* regulation in mouse skin cells, the human ECE18 ortholog is able to function in this context.

To investigate the functionality of hECE18 during eccrine gland development, we generated a humanized hECE18 knock-in (hECE18^KI^) by replacing the endogenous ECE18 enhancer of C57BL/6J mice with its human ortholog (Fig. 3*C* and *SI Appendix*, Fig. S6). hECE18^KI^ mice were born at expected Mendelian ratios and were viable and fertile. qRT-PCR analysis of *En1* expression in P2.5 volar skin revealed that hECE18^KI^ heterozygotes and homozygotes each had on average a 1.6-fold increase in *En1* relative to wild-type sibling controls (Fig. 3*D*). Given the evidence that mouse ECE18 is not required for *En1* expression in this context, competition with the endogenous mouse *En1* enhancers may help to explain why we did not observe a copy-dependent increase in *En1* expression between hECE18^KI^ homozygous and heterozygous knock-in mice. The effect of hECE18 on *En1* expression was restricted to the volar skin, and we did not observe ectopic expression in the haired dorsal limb (*SI Appendix*, Fig. S7*A*). Allele-specific analysis of *En1* transcription from the volar skin of FVB/N:C57BL/6J^wt^ and FVB/N:C57BL/6J^hECE18KI^ F1 hybrids revealed that hECE18-mediated up-regulation of *En1* occurs in *cis*, consistent with hECE18 acting as an enhancer of ectodermal *En1* in developing mouse volar skin (Fig. 3*E*).

Since hECE18 exerts a positive effect on ectodermal *En1* levels, we tested whether this enhancer could promote the specification of mouse eccrine glands. We did not observe a change in eccrine gland number in hECE18^KI^ mice (*SI Appendix*, Fig. S7*B*). This may reflect the relative attenuation of hECE18 potency in the mouse. Alternatively, since our data indicate that ECE18 is not required for *En1* regulation in mouse skin, the mouse may be insensitive to the human enhancer under endogenous conditions. Accordingly, we sensitized the number of volar eccrine glands formed to *En1* levels by crossing in a single copy of the *En1* knockout allele (En1^KO^) (35) and quantified the number of eccrine glands in the IFP of adult mice (Fig. 3*F*). Consistent with our previous studies (18, 20), En1^KO^ heterozygotes had fewer IFP eccrine glands than wild-type controls (*SI Appendix*, Fig. S7*C*). Strikingly, we found that hECE18 could partially rescue the En1^KO^ eccrine gland phenotype since En1^KO^/+; hECE18^KI^/+ mice carrying a copy of the human enhancer had on average 17% more IFP eccrine glands than En1^KO^ alone animals (En1^KO/+^; ^+/+^) (Fig. 3*G*). This result not only shows that the hECE18 enhancer can function in a developmental context to promote the formation of more eccrine glands but also implicates the up-regulation of ectodermal *En1* levels as the underlying mechanism for this effect.

## Discussion

**Fig. 4.**
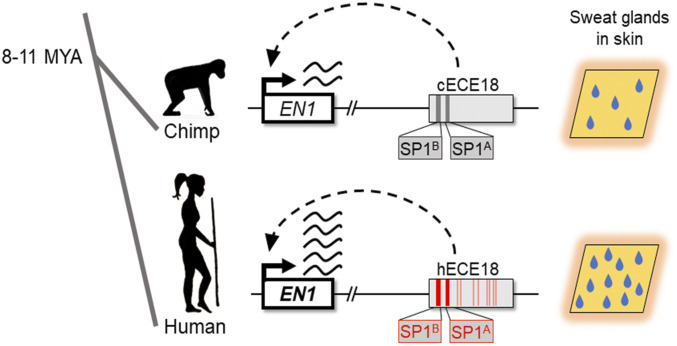
A genetic basis for the evolution of increased eccrine gland density in human skin. ECE18 is a developmental enhancer of the anthropoid *Engrailed 1* locus. Over the course of human evolution, the ECE18 enhancer accumulated multiple activity-enhancing single nucleotide mutations, particularly at the SP1 sites SP1^A^ and SP1^B^ (boxed and bold), that collectively induced the specification of more eccrine glands in human skin by potentiating developmental *EN1* expression. Derived human substitutions relative to other apes underlying higher activity of hECE18 are in red.

Natural selection has shaped many unique human attributes, but the genetic bases of these features are largely unknown. The findings of this study implicate a genetic and developmental mechanism harnessed in the evolution of one of the defining physiological adaptations of humans. That human ECE18 (hECE18) not only positively regulates *EN1* expression in cultured human skin cells but also in a developmental model of eccrine gland formation provides compelling evidence for a similar functionality for this enhancer in humans. Combined with the finding that human-specific mutations have increased the relative strength of hECE18, our study supports an evolutionary scenario in which a stronger hECE18 produced up-regulation of ectodermal *EN1* during development and a concomitant increase in the number of eccrine glands specified in human skin relative to that of other apes (Fig. 4).

An intriguing feature of hECE18 is that the accumulation of multiple point mutations scattered throughout the enhancer have collectively increased this element’s activity in keratinocytes. Derived human bp substitutions at conserved primate SP1 binding sites SP1^A^ and SP1^B^ appear to have played the greatest individual role in increasing hECE18 activity. Intriguingly, five of the remaining derived substitutions which contribute to increased hECE18 activity lie within the human accelerated region 2xHAR20. While future additional work will be required to tease apart the epistatic interactions between the mutations within and outside the HAR and to unravel the specific steps the HAR played in the evolution of higher eccrine gland density, it is worth noting that our findings directly implicate a bioinformatically defined HAR in the evolution of an adaptive human trait.

That hECE18 gains evolved in a stepwise manner suggests that this element could have contributed to evolutionary increases in the eccrine gland density of *Homo sapiens* at multiple points during human evolution. As such, our study raises the possibility of sustained selection on the multiple functional derived human mutations which together account for the increased activity of this enhancer relative to other primates. To understand this, we must parse out the order in which hECE18 variants evolved and the threshold at which hECE18 activity gains affect *EN1* and eccrine phenotypes. In terms of considering evolutionary mechanisms more broadly, our findings indicate that in addition to the traditional paradigm whereby human adaptive traits evolve as consequences of modifications to multiple components of a developmental program (36), sequential mutations in a single functional element that incrementally alter its activity is also a mechanism to produce gradual change in a trait over time.

A final point to consider is whether the evolution of hECE18 can by itself explain humans’ 10-fold increase in eccrine gland density over that of other apes. hECE18 produced a subtle increase in eccrine gland number in the *En1*-sensitized mouse knock-in. In light of the finding that ECE18 is not an essential part of the endogenous regulatory machinery controlling *En1* levels in the mouse, the mild effects of introducing the human enhancer may be reflective of an attenuated functionality of hECE18 in mouse skin. Determining whether there are associations between present-day hECE18 polymorphisms and variation in human eccrine gland density will help shed light on the relative importance of this enhancer to the specification of eccrine gland density in its endogenous, human context (*SI Appendix*, Fig. S3*G*). Future studies to comprehensively map the enhancers that control ectodermal *EN1* levels during eccrine gland specification and functional follow-up of the significance of derived human mutations therein may implicate additional *EN1*-dependent mechanisms that played a role in the evolution of human eccrine gland density. Candidates include ECE2, ECE8, ECE20, and ECE23 enhancers identified in this study. Moreover, because generalized eccrine glands are an innovation of Catarrhines, lineage-specific regulatory elements that are not conserved outside of this parvorder may have evolved to modulate *EN1* expression and eccrine phenotypes in humans and our closest primate relatives. Beyond *EN1*, another possibility is that other loci also contributed to the evolution of humankind’s elaborated eccrine gland density. This would be consistent with the finding that eccrine gland number is controlled by multiple genes, including *En1*, in mice (18). Indeed, we have previously shown that variation at another locus, *EDAR*, is responsible for population-level differences in eccrine gland density among modern humans (37). Distinguishing between these possibilities will require comprehensive delineation of the molecular pathways that regulate eccrine gland development. The mechanistic insight into how hECE18 is regulated provides a foundation to interrogate these pathways and to investigate the origins of other related traits, such as the generalization of eccrine glands to the nonvolar skin. Moreover, because of the importance of *En1* in the development of other organs and tissues, including derived human brain structures such as the cerebellum (38, 39), our findings may shed light on differences in the modulation of human *EN1* in these contexts and the etiology of other evolutionarily significant human phenotypes.

## Materials and Methods

### Identification of *En1* Candidate Enhancers for In Vivo Testing.

Conserved elements were called using phastCons (26, 40) from the alignment of placental mammals using the phastCons60way dataset. We restricted our scan to the *EN1* TAD defined by Hi-C in normal human cultured keratinocytes (NHEK) (Chr2: 118000000–118880000 [hg38]) since studies on the compartmentalization of the mammalian genome suggest that elements controlling expression of *EN1*, or indeed any locus, are likely to be located within this smaller interval (24, 25). We called 209 conserved elements based on strings of at least 50 bps of consecutive high phastCons (26) values using an empirically determined cutoff of ≥98% probability of being conserved, excluding coding exons and allowing for short gaps of missing data. We prioritized 41 of these conserved elements based on overlap with the following published epigenomic marks: DNase hypersensitivity peaks from embryonic mouse limbs (27), Capture-C peaks from mouse embryonic limbs (28), H3K27Ac ChIP-seq peaks from mouse embryonic limbs (27), H3K27Ac ChIP-seq peaks from NHEK cells (27), coordinates of known HARs (29–31), and DEAF1 ChIP-seq peaks (20). Using each of these 41 priority elements as a kernel, we expanded these genomic regions to 1,000 to 1,500 bps because known enhancers tend to be hundreds of base pairs long, collapsing our list to 23 ECEs for functional testing.

### Mice.

CD1 (Crl:CD1) timed pregnant mice and FVB/NCrl mice were purchased from Charles River Laboratories. C57BL/6J mice were purchased from the Jackson Laboratories. En1^KO^ mice were generated in the laboratory of Alexandra Joyner (35) and were obtained from the laboratory of Susan Dymecki. En1^KO^ mice were bred onto C57BL/6NTac (Taconic Biosciences) for at least 10 generations. All experiments were performed in accordance with approved Institutional Animal Care and Use Committee (IACUC) protocols.

### Generation of Lentiviral-Mediated Transient Transgenic Mice.

The generation of lentivirus-mediated transient transgenic mice was performed as previously described (32, 41). In brief, each ECE or ECE fragment was cloned into the Stagia3 lentiviral enhancer reporter vector, upstream of the minimal TATA box promoter and eGFP reporter cassette (33, 34). Primers used for cloning are reported (*SI Appendix*, Table S1). A high-titer lentivirus for each construct was prepared in HEK293T cells according to established protocols using a second-generation packaging system (gift from Connie Cepko, Harvard Medical School, Boston, MA). The transfection of HEK293T cells was carried out using polyethylenimine (Polysciences, Inc.). Following concentration, a lentivirus for each tested DNA element was mixed in a 7:1 ratio with a control virus expressing mCherry under a ubiquitous promoter (modified FUtdTW in which tdTomato was replaced with an mCherry expression cassette was a gift from Connie Cepko, Harvard Medical School, Boston, MA). The expression of mCherry was used to identify successfully transduced mice at harvest. A virus for each tested element in this study, mixed with FUtdTW, was injected into the amniotic cavity of embryonic day (E) 9 CD1/NCrl mouse embryos (Charles River Labs). Injections were carried out under ultrasound guidance using the Vevo 2100 ultrasound imaging system (Visualsonics) equipped with a 35 to 50 MHz mechanical transducer as described previously (32, 41). At E9, lentiviral particles stably integrate into the genomes of cells of the single layer mouse ectoderm but cannot pass beyond the underlying basement membrane, allowing for ectoderm-specific generation of viral transgenic mice (32). All layers of the skin, including the basal keratinocyte layer, derive from the single layer ectoderm at E9. At least six embryos were injected per tested DNA element. All survival surgeries were carried out in accordance with approved IACUC protocols.

On P2.5, pups were euthanized, and both forelimbs and both hindlimbs were processed for expression of eGFP in mCherry-positive animals. At this stage, *En1* is expressed throughout basal keratinocytes of the distal volar skin, is focally up-regulated in eccrine gland placodes of the IFP, and is critical for specifying IFP eccrine gland number (Fig. 1*A*) (18). This spatial pattern is not recapitulated by other transcripts encoded in the TAD. Positive elements were called based on the observation of multiple eGFP-positive clones in at least three individual transduced mice. Of note, since viral integration into the infected cell genome occurs independently and at random, each clone of positive cells constitutes an individual transgenic event.

### In Situ Hybridization, Immunohistochemistry, and Imaging.

Whole forelimb and hindlimb autopods (the distal segment of the limb) were embedded in OCT (Tissue Tek) and cryosectioned at a thickness of 10 to 12 µm. *En1* in situ hybridization was performed as previously described (18). HRP (horseradish peroxidase)/DAB (3,3-diaminobenzidine) immunostaining was performed as follows. Tissue sections were fixed in 4% paraformaldehyde (PFA) and then washed in phosphate-buffered saline (PBS). Endogenous peroxidase was blocked using 0.3% hydrogen peroxide. The tissue was blocked in PBST (PBS + 0.1% Tween) + 10% normal donkey serum before incubating in chicken anti-GFP primary antibody (1:2,000, Jackson). After washing, samples were incubated with biotin-SP–conjugated rabbit anti-chicken secondary antibody (1:250, Jackson ImmunoResearch). Samples were washed and incubated in Vectastain ABC reagent (Vector Laboratories). Enzymatic detection was carried out using the DAB peroxidase substrate kit (Vector Laboratories) according to manufacturer’s instructions. Images were acquired on a Leica DM5500 B microscope equipped with a Leica DEC500 camera.

### Bidirectional Luciferase Vector and Luciferase Assays.

Nonhuman primate genomic DNA samples were obtained from Christopher Brown (Perelman School of Medicine at the University of Pennsylvania, Philadelphia, PA). A domestic female cat (*Felis catus*), named Phila, served as the source of genomic DNA. All cloned genomic fragments were sequence verified against the current genome assemblies,

The bidirectional luciferase lentiviral reporter was built based on refs. 42 and 43 and by using Stagia3 as a backbone (*SI Appendix*, Fig. S3*B*). Briefly, the different ECE18 orthologs and the various fragments described in this study (*SI Appendix*, Fig. S3*C*) were cloned upstream of a minimal TATA box promoter and upstream of the Firefly luciferase reporter gene into the bidirectional luciferase lentiviral vector and using the following primers (*SI Appendix*, Table S2). Lentivirus production and skin cell transduction were carried out as previously described (20, 44). Cells were harvested at 72 h post-transduction, and Firefly and Renilla (for normalization) luciferase activities were measured using the Dual-Luciferase Reporter Assay System (Promega). The assay was performed using the SpectraMax i3x (Molecular Devices). Experiments were done independently at least three times in biological triplicate each time. Mutagenesis of ECE18 orthologs was carried out using standard site-directed mutagenesis protocols, and primers are reported in *SI Appendix*, Table S3.

### In Silico Motif Discovery Analysis.

SP1 DNA-binding sites were first identified by looking at conserved motifs across the ECE18 sequence and using the Evolutionary Conserved Regions (ECR) browser with default parameters (TRANSFAC professional V10.2 library) (45). The prediction of SP1 DNA-binding motifs at the selected sites was confirmed using JASPAR 2018, and affinity scores were obtained from the JASPAR 2018 database (Matrix identification: MA0079.3) (46).

### Cell Culture, Transfection, and Transduction.

Primary mouse keratinocytes were isolated and cultured as previously described with minor modifications (47, 48). Briefly, CD1 P0 pups were euthanized, and then back skin was removed from the body and incubated over night with 0.25% trypsin-ethylenediaminetetraacetic acid (EDTA; Gibco) to detach the epidermis from the dermis. The next day, the epidermis was collected in a conical tube, and the keratinocytes were dissociated by mechanical shearing by nutation at a slow speed for 45 min at 4 °C. Cells were plated and maintained using Keratinocyte Serum Free Medium (Gibco) containing 45 µM calcium, 4% of chelex-treated fetal bovine serum (HyClone), 10 ng/mL mouse epidermal growth factor (Corning), 50 µg/mL bovine pituitary extract (Gibco), and streptomycin/penicillin (Sigma).

GMA24F1A human keratinocytes were a gift from Howard Green (Harvard Medical School, Boston, MA) and were previously described (23). GMA24F1A cells are a clonal line of human keratinocytes that express *EN1* and have basal keratinocyte characteristics, which we confirmed by the expression of the signature basal keratin *Keratin 14* (*KRT14*) and by the expression of *EN1*.

HEK293T cells were obtained from the laboratory of Connie Cepko (Harvard Medical School, Boston, MA). The transfection of HEK293T cells was carried out using polyethylenimine (Polysciences, Inc.). We used a second-generation packaging system to generate the lentiviruses used in this study (gift from Connie Cepko, Harvard Medical School, Boston, MA). All viruses were produced in HEK293T cells according to established protocols. The transduction of GMA24F1A cells and primary mouse keratinocytes was carried out as previously described (20, 44). Packaging plasmid psPAX2 was a gift from Didier Trono (École Polytechnique Fédérale de Lausanne, Lausanne, Switzerland, Addgene plasmid number 12260), and envelope plasmid pCL-VSV was a gift from Connie Cepko (Harvard Medical School, Boston, MA).

### Chromatin Immunoprecipitation and qPCR.

ChIP-qPCR was performed as previously described (20). In brief, GMA24F1A cells were cultured in 150 mm dishes and harvested for immunoprecipitation using the EZ-Magma ChIP HiSens Kit (EMD Millipore) according to the manufacturer’s instructions. DNA was cross-linked by adding formaldehyde (1% final concentration) for 10 min at room temperature. After cell lysis, the chromatin was sheared using a Covaris M220 ultrasonicator, and the time was optimized to get chromatin fragments between 100 and 500 bps. Immunoprecipitation was carried out using 1/125 SP1 Antibody (ab13370, Abcam) or 1 ug of normal rabbit IgG antibody (Sigma-Aldrich). Input and immune-precipitated samples were purified using a Qiagen PCR purification kit. qPCR was done using the Power SYBR PCR master mix (Thermo Fisher). SP1 occupancy of a given region after the immunoprecipitation was determined as the percentage of enrichment over the input using the equation 100 × 2Ct^(input)^^–^^Ct(IP)^ and is represented in all graphs as enrichment of SP1 or IgG. The primers used for qPCR are reported in *SI Appendix*, Table S4. The ChIP-qPCR experiment was performed three times, and each reported value represents the average across three technical qPCR replicates for that biological sample in a single experiment.

### Generation and Maintenance of Mouse ECE18 Knockout (mECE18^del^) and hECE18^KI^ Mice.

For specific details of targeting design, strategy and validation, screening of founder animals, and establishment of mutant lines see *SI Appendix*, Fig. S5 (mECE18^del^) and *SI Appendix*, Fig. S6 (hECE18^KI^). In brief, pairs of gRNAs were designed using the online tool http://crispr.mit.edu/. Guides were tested to generate deletion of mouse ECE18 in vitro in NIH 3T3 cells. For the generation of hECE18^KI^, a single-stranded repair template containing the hECE18 genomic sequence flanked by mouse genomic sequence homology arms was synthesized, sequence confirmed, and purchased from Genewiz. The reported gRNAs which were confirmed to target mouse ECE18 in vitro were used to generate genome edited mice. gRNA selection, generation, and in vitro testing were performed by the Perelman School of Medicine (PSOM) CRISPR Cas9 Mouse Targeting Core. Validated gRNAs along with Cas9 RNA and a hECE18 repair template were microinjected into the cytoplasm of C57BL/6J one cell embryos by the PSOM Mouse Transgenic and Chimeric mouse facility. The hECE18 knock-in fragment was confirmed by Sanger sequencing. All procedures were performed in accordance with approved IACUC protocols.

### Southern Blot Analysis.

Genomic DNA was isolated from mouse tail biopsies using tail lysis buffer (10 mM Tris pH: 8.0, 100 mM NaCl, 10 mM EDTA pH: 8.0, and 0.5% sodium dodecyl sulfate [SDS])/Proteinase K and extracted by a Phenol:Chloroform:Isoamyl alcohol method. In brief, 10 μg of genomic DNA was digested with KpnI-HF and NotI-HF (New England Biolabs) overnight at 37 °C, followed by heat inactivation for 20 min at 65 °C. Digested genomic DNA was run on 1% agarose gels and transferred to a 0.45 μm hybridization nitrocellulose filter (Millipore). The membrane was hybridized with a ^32^P-labeled specific DNA probe (*SI Appendix*, Fig. S6*E*). The probe was labeled using a Nick Translation Kit (Roche) and [α-32P] deoxycytidine-5′triphosphate (PerkinElmer). The membrane was washed with 0.1× saline sodium citrate (SSC) buffer (1× SSC is 150 mM NaCl, 15 mM sodium citrate) and 0.1% SDS at 55 °C. Membrane was exposed to a phosphor screen and was imaged on the Amersham Typhoon Biomolecular Imager (GE).

### Long-Range PCR and DNA Digestion.

Genomic DNA fragments of interest were PCR-amplified using Q5 polymerase (New England Biolabs) (for primers, see *SI Appendix*, Fig. S6*F*) and purified using gel extraction (Qiagen). PCR products were digested using NotI (New England Biolabs) (*SI Appendix*, Fig. S6*F*).

### dCas9-KRAB Repression of hECE18.

gRNAs were designated using the online Integrated DNA Technologies DNA tool (https://www.idtdna.com/) (Cr1: 5′-GAT​TTT​CAT​TTC​CTG​TGT​TA-3′ and Cr2: 5′-TCT​TTT​CTG​TTT​ACC​GGG​GA-3′). pLV hU6-sgRNA hUbC-dCas9-KRAB-T2a-GFP, which encodes dCas9-KRAB fusion was a gift from Charles Gersbach (Addgene plasmid number 71237; http://n2t.net/addgene:71237; Research Resource Identification: Addgene_71237) (49). gRNAs were cloned into the plasmid as previously described (49). Lentiviral production was done using the HEK293T cells line, and GMA24F1A cell transduction was performed as previously described (20) with modifications. Briefly, low confluency GMA24F1A cells were transduced. Then, cells were harvested 5 d post-transduction when between 90 to 100% of the cells expressed the reporter GFP protein, indicating that most cells integrated the vector producing the gRNA and the dCas9-KRAB protein. Two independent experiments were carried out in biological triplicates each time.

### RNA Extraction and qRT-PCR.

Total RNA was isolated using TRIzol (Life Technologies). Next, we cleaned up the RNA using the RNeasy Mini Kit (Qiagen) according to the manufacturer’s instructions. *En1* total expression was determined as previously described (20) and by using the following primers (*SI Appendix*, Table S4). Assays were performed in biological triplicates consisting of at least six pooled ventral forelimb skins from each genotype at P2.5. Complementary DNA (cDNA) was generated using SuperScript III (Thermo Fisher) following the manufacturer’s instructions. qRT-PCR was done using the Power SYBR PCR master mix (Thermo Fisher) and in technical triplicates each time.

### Allelic Discrimination Assay.

*En1* allelic expression assays were performed as previously described (18). Briefly, ventral forelimb skin consisting of the region containing the five volar footpads and intervening IFP were dissected from P2.5 F1 C57BL/6J:FVB/N hybrid mice, and RNA was extracted. Amplification of cDNA and genomic DNA products was done using the following primers: *En1* forward 5′-GAG​CAG​CTG​CAG​AGA​CTC​AA-3′ and *En1* reverse 5′-CTC​GCT​CTC​GTC​TTT​GTC​CT-3′. *En1* allelic expression was determined by the relative expression of C57BL/6J (hECE18^KI^) or (mECE18^del^) versus FVB/N as distinguished at the genotype at rs3676156. Allelic expression data were analyzed using the sequencing-based QSVanalyzer software (50). Sequencing was carried out using the aforementioned *En1* forward primer in technical triplicate for each sample. cDNA was obtained from biological triplicates consisting of six or eight pooled ventral forelimb skins for each genotype.

### Quantification of Eccrine Glands in Forelimb IFP Skin.

Quantification of mouse IFP eccrine gland numbers was performed as previously described (37). In brief, 3 to 4 wk old mice were euthanized, and the mouse ventral forelimb skin was dissected for dissociation in Dispase II (Roche) to isolate epidermal whole mount preparations. Eccrine gland ducts remain attached to the epidermis after Dispase II treatment, allowing quantification of eccrine gland number based on the number of ducts emanating from the epidermis. Whole mount epidermal preparations were stained with Nile Blue (Sigma-Aldrich) and Oil Red O (Sigma-Aldrich) to visualize volar skin appendages (hair follicles and eccrine glands) and hair follicle–associated sebaceous glands, respectively. IFP eccrine gland numbers were quantified by averaging the number of glands across the left and right forelimb in epidermal preparations from each animal.

### Statistical Analysis.

Statistical analysis was performed using either ordinary one-way ANOVA followed by Tukey multiple comparisons correction with a single pooled variance on the means for each dataset or student’s unpaired *t* test (two tailed) using GraphPad Prism version 7.04 for Windows (http://www.graphpad.com/).

### Genome Alignments.

Visualiziation of genome alignments and genomic annotations were generated using the UCSC Genome Browser database (https://genome.ucsc.edu) (51).

## Supplementary Material

Supplementary File

## Data Availability

All study data are included in the article and/or *SI Appendix*.
