# Supplementary material for: Repeated mutation of a developmental enhancer contributed to human thermoregulatory evolution
Source: Proc Natl Acad Sci U S A. 2021 Apr 13;118(16):e2021722118. doi: 10.1073/pnas.2021722118 (PMC8072367; doi:10.1073/pnas.2021722118)
Supplement: Supplementary File [file pnas.2021722118.sapp.pdf]

PNAS
www.pnas.org

Supplementary Information for
Repeated mutation of a developmental enhancer contributed to the human thermoregulatory evolution

Daniel Aldea, Yuji Atsuta, Blerina Kokalari, Stephen Schaffner, Rexxi D. Prasasya, Adam Aharoni, Heather L. Dingwall, Bailey Warder, Yana G. Kamberov

Yana G. Kamberov
Email: yana2@pennmedicine.upenn.edu

This PDF file includes:

Figures S1 to S7
Tables S1 to S4
SI References

A List of Conserved Elements within En1 TAD in mm10

Legend:
1. DNase I hypersensitivity from mouse limbs at E11.5
2. C-capture from mouse limbs at E13.5
3. H3K27Ac from Normal human Epidermal keratinocytes (NHEK)
4. Deaf1 ChIP-seq peaks from culture human keratinocytes
5. Human Accelerated Regions (HARs)
6. Conserved element prioritized

N°	Chr	Coordinates		1	2	3	4	5	6
1	chr1	120591009	120591104						
2	chr1	120598188	120598297						
3	chr1	120598340	120598390						
4	chr1	120600010	120600554						■
5	chr1	120602217	120602283			■			■
6	chr1	120602295	120602437			■	■		■
7	chr1	120602440	120602556						
8	chr1	120602684	120602753						
9	chr1	120602790	120602841						
10	chr1	120602923	120603031						
11	chr1	120605018	120605127			■			■
12	chr1	120607550	120607619						■
13	chr1	120607712	120607777						■
14	chr1	120607866	120607987						■
15	chr1	120615893	120615948						
16	chr1	120621495	120621577						■
17	chr1	120623207	120623409						
18	chr1	120623411	120623516						
19	chr1	120624808	120624874						
20	chr1	120625309	120625433						
21	chr1	120627393	120627472	■					
22	chr1	120643993	120644048	■					
23	chr1	120644052	120644132	■					
24	chr1	120646765	120646904						
25	chr1	120650281	120650345	■					
26	chr1	120659671	120659832	■					
27	chr1	120671678	120671765			■			
28	chr1	120680894	120680943						
29	chr1	120686909	120686985	■					
30	chr1	120692870	120692942						■
31	chr1	120692948	120693060						■
32	chr1	120693066	120693155						■
33	chr1	120703968	120704057						
34	chr1	120715353	120715407	■					■
35	chr1	120715447	120715672	■					■
36	chr1	120727572	120727631	■					
37	chr1	120727748	120727798	■					
38	chr1	120729086	120729233						
39	chr1	120729261	120729316						
40	chr1	120734650	120734706	■					
41	chr1	120741558	120741623	■					
42	chr1	120749344	120749394	■					
43	chr1	120749404	120749481	■					
44	chr1	120753149	120753213						
45	chr1	120753250	120753299						
46	chr1	120754341	120754474						
47	chr1	120754476	120754534						
48	chr1	120754586	120754635						
49	chr1	120755496	120755549						
50	chr1	120757086	120757184	■		■			■
51	chr1	120757186	120757349	■		■			■
52	chr1	120760999	120761049	■					■
53	chr1	120770402	120770496	■					
54	chr1	120770499	120770564	■					
55	chr1	120795644	120795727	■					
56	chr1	120795732	120795785	■					
57	chr1	120795790	120795912	■					
58	chr1	120795919	120796012	■					
59	chr1	120796024	120796139	■					
60	chr1	120801934	120801991						
61	chr1	120811068	120811128						
62	chr1	120816565	120816680						
63	chr1	120817814	120817864						
64	chr1	120825152	120825217						
65	chr1	120831635	120831712						
66	chr1	120842548	120842630	■					■
67	chr1	120859206	120859284	■					
68	chr1	120859288	120859523	■					
69	chr1	120859671	120859747	■					
70	chr1	120868488	120868554						
71	chr1	120868560	120868653						
72	chr1	120868843	120868969						
73	chr1	120872585	120872694						
74	chr1	120872907	120873009						
75	chr1	120883400	120883465						
76	chr1	120883467	120883526						
77	chr1	120883553	120883664						
78	chr1	120883824	120883931						
79	chr1	120886022	120886086						
80	chr1	120886126	120886179						
81	chr1	120886245	120886526						
82	chr1	120887118	120887234						
83	chr1	120910913	120911071	■					■
84	chr1	120912165	120912320	■					
85	chr1	120912323	120912407	■					
86	chr1	120912409	120912498	■					
87	chr1	120912527	120912591	■					
88	chr1	120941703	120941870						
89	chr1	120941874	120942147						
90	chr1	120951771	120951826	■					
91	chr1	120951917	120951967	■					
92	chr1	120953043	120953094						
93	chr1	120953191	120953282						
94	chr1	120961812	120961864	■					■
95	chr1	120961945	120962094	■					■
96	chr1	120962096	120962173	■					■
97	chr1	120964202	120964331	■					■
98	chr1	120964433	120964513	■					■
99	chr1	120970137	120970243			■			■
100	chr1	120983669	120983722						
101	chr1	121005936	121006022	■					■
102	chr1	121006028	121006114	■					■
103	chr1	121012716	121012903	■					
104	chr1	121026894	121026949						
105	chr1	121026972	121027042						
106	chr1	121032467	121032517						
107	chr1	121046637	121047008						
108	chr1	121047045	121047283						
109	chr1	121047309	121047395						
110	chr1	121050621	121050692	■					■
111	chr1	121051324	121051472	■					■
112	chr1	121051480	121051551	■					■
113	chr1	121054103	121054259						■
114	chr1	121056740	121056816						
115	chr1	121069303	121069365						
116	chr1	121069395	121069465						
117	chr1	121070294	121070348						
118	chr1	121075119	121075172						
119	chr1	121087368	121087417						
120	chr1	121087894	121088031						
121	chr1	121088063	121088164						
122	chr1	121088168	121088241						
123	chr1	121096210	121096270						
124	chr1	121096963	121097041	■		■			■
125	chr1	121097211	121097366	■		■	■	■	■
126	chr1	121097372	121097600	■		■	■	■	■
127	chr1	121097604	121097775	■		■	■	■	■
128	chr1	121099214	121099268						
129	chr1	121101382	121101434						
130	chr1	121101438	121101536						
131	chr1	121101604	121101740						
132	chr1	121117042	121117138						
133	chr1	121117151	121117228						
134	chr1	121117236	121117330						
135	chr1	121117361	121117421						
136	chr1	121117437	121117495						
137	chr1	121121375	121121452						
138	chr1	121121530	121121582						
139	chr1	121122653	121122704						
140	chr1	121122710	121122899						
141	chr1	121122902	121123061						
142	chr1	121123064	121123119						
143	chr1	121123143	121123238						
144	chr1	121127101	121127184						
145	chr1	121130584	121130641						
146	chr1	121136381	121136552						
147	chr1	121136582	121136637						
148	chr1	121143058	121143116						
149	chr1	121143145	121143208						
150	chr1	121155017	121155244						■
151	chr1	121168639	121168691						
152	chr1	121176298	121176377		■				
153	chr1	121177154	121177468		■	■			■
154	chr1	121180887	121180980	■	■				■
155	chr1	121180992	121181047	■	■				■
156	chr1	121224243	121224401			■			■
157	chr1	121226826	121227009						
158	chr1	121227011	121227094						
159	chr1	121227169	121227228						
160	chr1	121227234	121227766						
161	chr1	121237491	121237739						
162	chr1	121237744	121237855						
163	chr1	121254210	121254413						
164	chr1	121254791	121254850						
165	chr1	121290954	121291077						
166	chr1	121291134	121291207						
167	chr1	121298130	121298192						
168	chr1	121304358	121304418						
169	chr1	121304448	121304541						
170	chr1	121307887	121307942						
171	chr1	121308031	121308154						
172	chr1	121308271	121308340						
173	chr1	121314738	121314792						
174	chr1	121344380	121344480						
175	chr1	121344484	121344538						
176	chr1	121355789	121355884						
177	chr1	121355892	121355949						
178	chr1	121355955	121356029						
179	chr1	121370913	121370991						
180	chr1	121371381	121371524						
181	chr1	121371531	121371666						
182	chr1	121371703	121371754						
183	chr1	121371793	121371860						
184	chr1	121371918	121371983						
185	chr1	121379299	121379443						
186	chr1	121379500	121379604						
187	chr1	121381958	121382018						
188	chr1	121388490	121388588						
189	chr1	121388593	121388648						
190	chr1	121388681	121388744						
191	chr1	121390182	121390306						
192	chr1	121390308	121390390						
193	chr1	121390500	121390697						
194	chr1	121390893	121391056						
195	chr1	121391107	121391376						
196	chr1	121392525	121392914						
197	chr1	121392970	121393078						
198	chr1	121393389	121393530						
199	chr1	121393678	121393972						
200	chr1	121393976	121394027						
201	chr1	121394935	121394998	■					■
202	chr1	121395002	121395189	■					■
203	chr1	121399546	121399678						
204	chr1	121399711	121399770						
205	chr1	121400200	121400326						
206	chr1	121400333	121400389						
207	chr1	121407825	121407885						
208	chr1	121407887	121408024						
209	chr1	121408036	121408119						

B List of En1 Candidates Enhancers (ECEs) tested *in vivo*

	Coordinates (mm10)	1	2
ECE1	120600025-120600904	4	
ECE2	120601976-120602486	5,6	*En1* promoter
ECE3	120604762-120605416	11	
ECE4	120607302-120608054	12,13,14	
ECE5	120621472-120622204	16	
ECE6	120692742-120693280	30,31,32	
ECE7	120714773-120716154	34,35	
ECE8	120756823-120757766	50,51	
ECE9	120760766-120761883	52	
ECE10	120842070-120843227	66	
ECE11	120910517-120911428	83	
ECE12	120961285-120962463	94,95,96	
ECE13	120963768-120964907	97,98	
ECE14	120969875-120970619	99	
ECE15	121005143-121006490	101,102	
ECE16	121050854-121051942	110,111,112	
ECE17	121053456-121054528	113	
ECE18	121096764-121097826	124,125,126,127	**This study**
ECE19	121154371-121155567	150	
ECE20	121176848-121178300	153	
ECE21	121179419-121181061	154,155	
ECE22	121223853-121224693	156	
ECE23	121394405-121395702	201,202	

Legend:

1. Conserved element contained within the ECE tested *in vivo* (see identification number in Extended Data 1a)

2. Reporter expression in *En1* positives domain in mouse ventral limb at P2.5

Positive

Negative

C Human derived substitutions relative to chimp/gorilla or to chimp

	Human derived substitutions relative to chimp/gorilla	% of the ECE	Human derived substitutions relative to chimp	% of the ECE
ECE2	5	0.93	12	2.24
ECE8	5	0.51	11	1.13
**ECE18**	13	**1.33**	24	**2.46**
ECE20	21	1.51	28	2.01
ECE23	7	0.81	13	1.50

Fig. S1. Genomic location and characteristics of conserved elements and *Engrailed 1* Candidate Enhancers (ECE). (A) Coordinates (mm10) of 209 conserved elements within the EN1 TAD identified by phastCons across placental mammals. Each element has a corresponding identifier (N). Criteria used to prioritize conserved elements: overlap with published datasets of epigenomic marks associated with enhancer presence (columns 1-3) **(1, 2)**; overlap with DEAF1 ChIP-seq peaks, which is a transcription factor we recently showed positively regulates Engrailed 1 in human and mouse keratinocytes (column 4) **(3)**; overlap with annotated human accelerated regions (HARs) (column 5) **(4–6)**. Overlap is indicated in red. Prioritized conserved elements which were used as kernels for ECEs are highlighted in orange in column 6. **(B)** Genomic coordinates

(mm10) of top 23 ECEs tested in mouse transgenic assays. Conserved elements (N) contained within each ECE are listed in column 1. ECEs that induced eGFP-positive clones within the En1-positive expression domain (basal keratinocytes of volar limb) are indicated by orange color in column 2. **(C)** Human derived substitutions relative to chimp/gorilla, or to chimp are listed as absolutes numbers or percentages calculated with respect to the total length in nucleotides of each positive ECE. ECE18 is boxed in red**.**

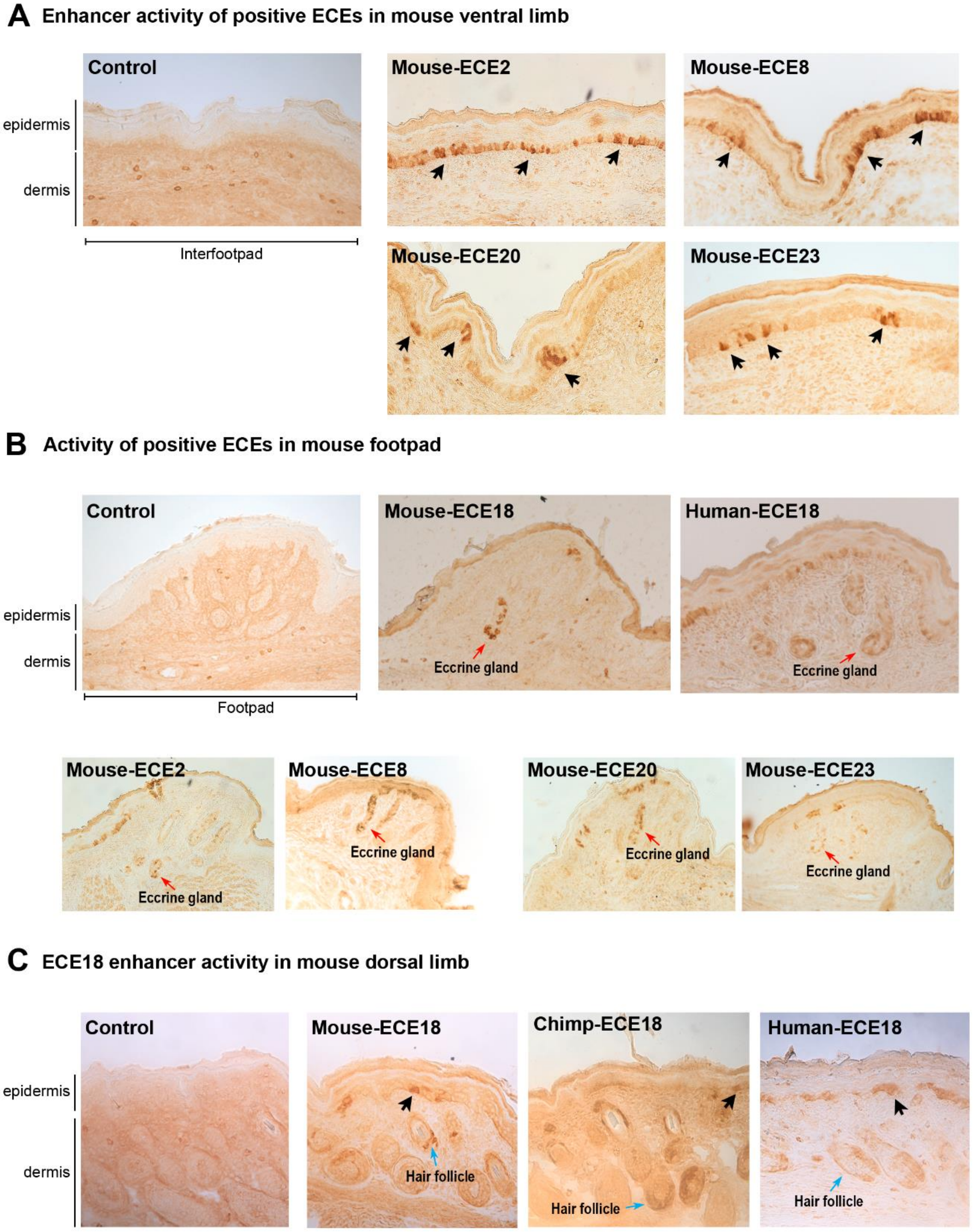

Fig. S2. Enhancer activity of positives ECEs in transgenic mouse skin. In all panels representative images of GFP antibody-stained sections of limbs from lentiviral-mediated

transgenic mice. Cross-sections of stained ventral mouse limbs **(A)**, and footpads **(B)** at P2.5 stage of positives ECEs identified in this study are shown. The eccrine glands of the footpads in **(B)** (red arrow) are undergoing differentiation as evidenced by their invagination into the dermal layer. **(C)** Representative images of P2.5 dorsal distal autopod skin from mouse, chimpanzee and human ECE18 lentiviral-mediated transgenic mice. The large hair follicles (blue arrow) which like eccrine glands derive from basal keratinocytes during development **(7, 8)**, and are characteristic of dorsal skin are also present. eGFP expression was detected by anti-GFP antibody and HRP/DAB coupled immunohistochemistry. GFP positive clones (black arrow). Control images from transgenic skin infected with lentivirus carrying minimal promoter and eGFP-reporter cassette alone.

A **Multiz alignment of placental mammals centered on human ECE18**

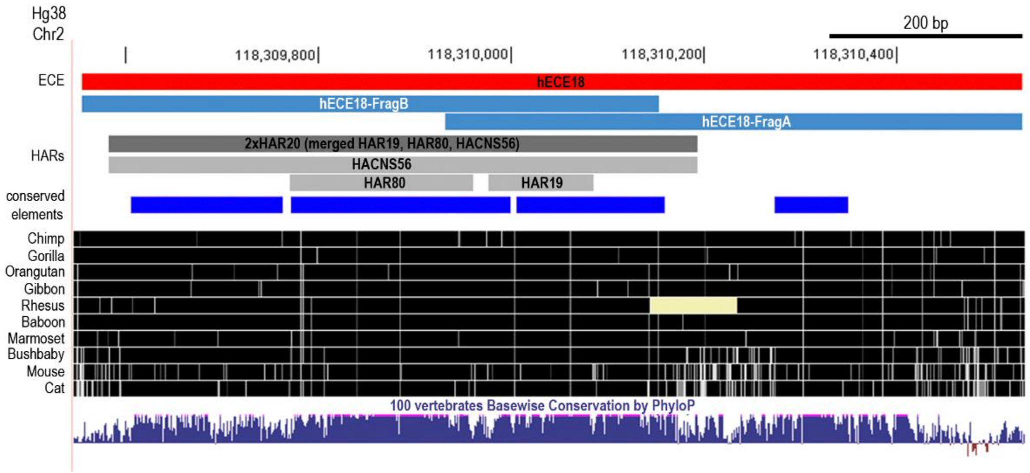

B **Bidirectional luciferase lentiviral vector**

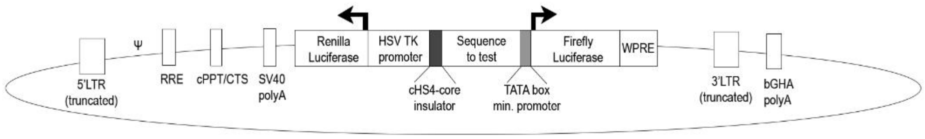

C **Coordinates of ECE18 homologs and others fragments tested *in vitro***

Species (build)	Chr	ECE18 Coordinates	ECE18-FragA Coordinates	ECE18-FragB Coordinates	2xHAR20 Coordinates	HAR19 Coordinates	HAR80 Coordinates
Mouse (mm10)	Chr1	121096764-121097826	121096764-121097440	-	-	-	-
Human (hg38)	Chr2	118309555-118310531	118309932-118310531	118309555-118310154	118309583-118310193	118309977-118310085	118309771-118309961
Chimp (panTro6)	Chr2B	4758321-4759295	4758698-4759295	-			
Gorilla (gorGor4)	Chr2B	-	7012616-7013213	-			
Macaque (rheMac10)	Chr12	12819060-12820038	12819060-12819658	-			
Marmoset (calJac3)	Chr6	92356458-92357434	-	-			
Bushbaby (otoGar3)	GL873578	3870683-3871687	-	-			
Cat (felCat9)	C1	122367966-122368951	122367966-122368592	-			

D **Human ECE18^{FragA} enhancer activity in mouse limb**

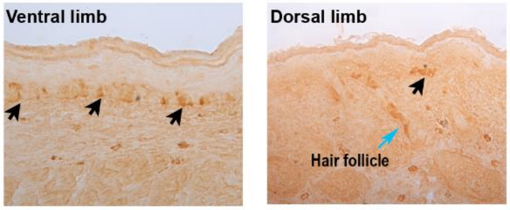

E **Activity of ECE18^{FragA} homologs**

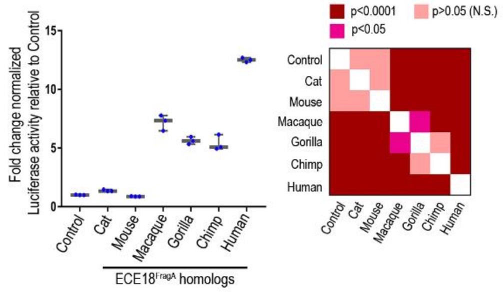

F Allele frequencies of human variants

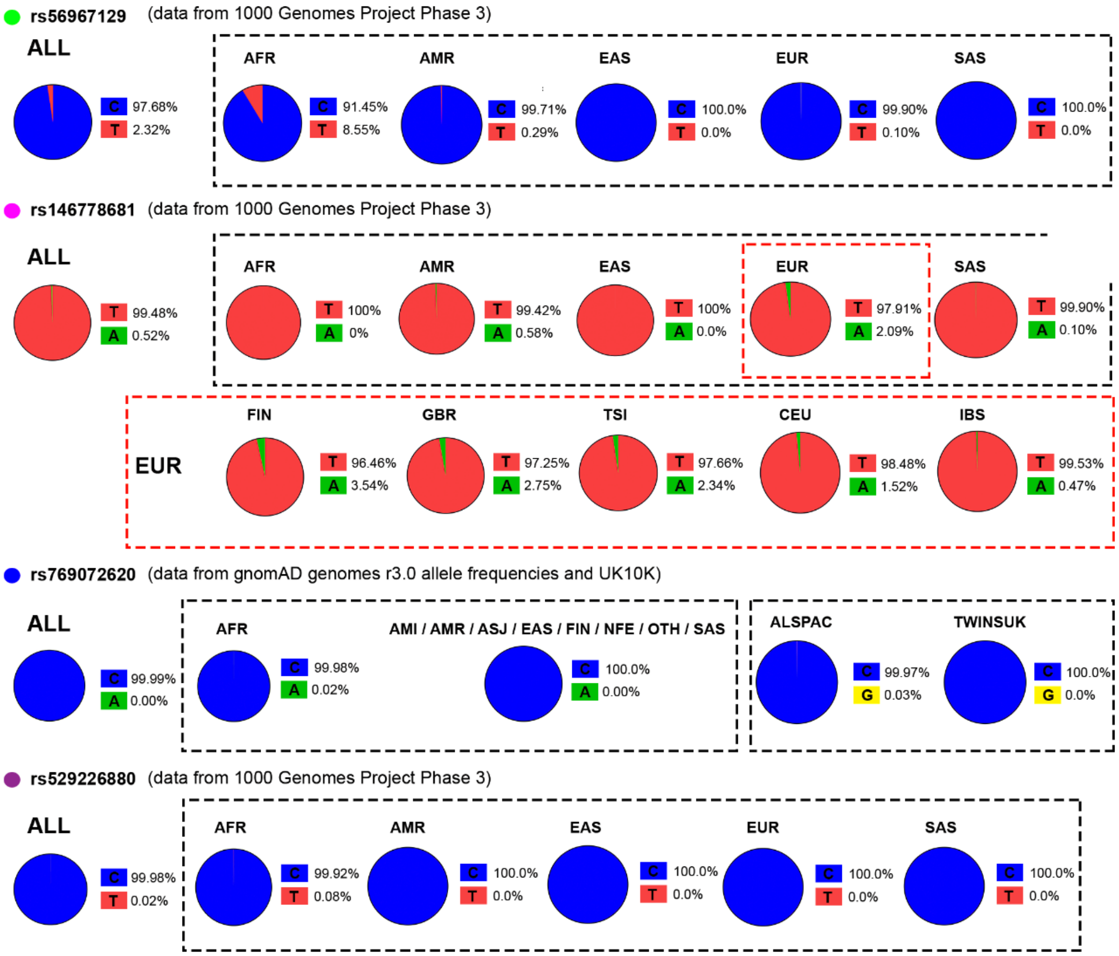

Legend:

AFR: African **AMR:** American **AMR:** American **EAS:** East Asian **EUR:** European **SAS:** South Asian
CEU: Utah resitents with Northern and Western European ancestry **FIN:** Finnish from Finland **TSI:** Toscani in Italy
GRB: British in England and Scotland **IBS:** Iberian populations in Spain **AMR:** Latino/Admixed americano
AMI: Amish **ASJ:** Ashkenazi Jewish **NFE:** Non-finish European
ALSPAC: The Avon Longitudinal Study of Parents and Children (UK10K COHORT ALSPAC) **OTH:** Other
TWINSUK: Twin registry of 11,000 identical and non-identical twins (UK10K COHORT TWINSUK)

G Effect of humans polymorphic variants on ECE18^{FragA} activity

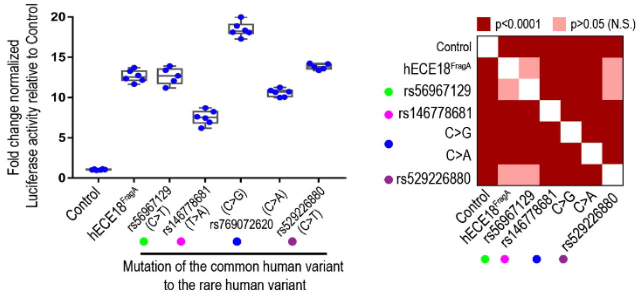

H Activity of chimeric ECE18

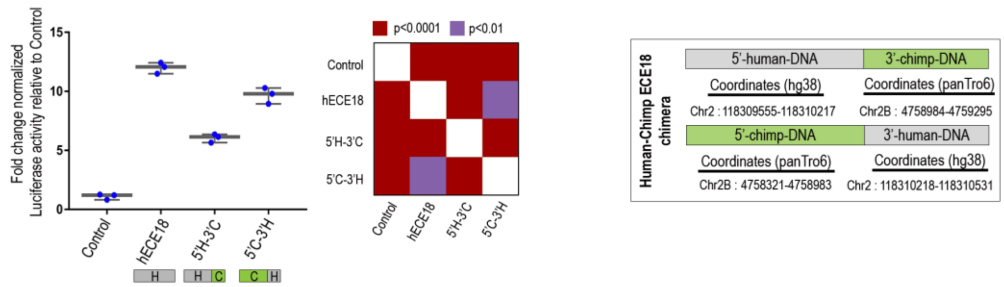

I Coordinates of human ECE18^{FragA} derived variants

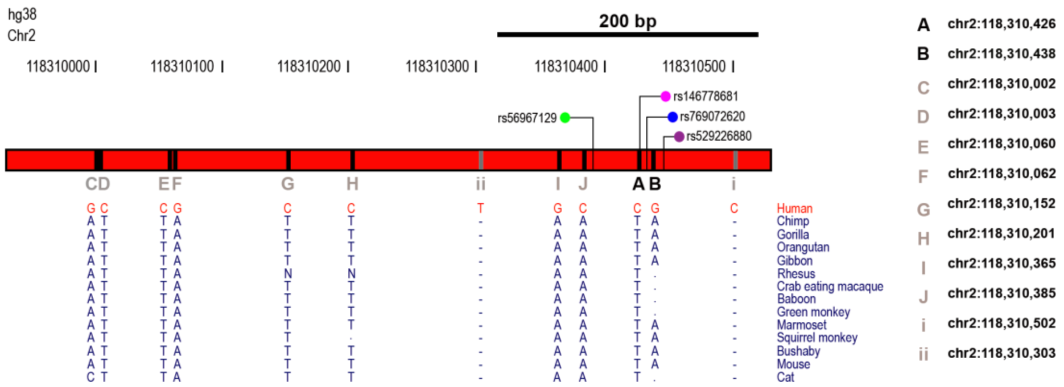

J Effect of deleting human specific insertions on hECE18^{fragA} activity

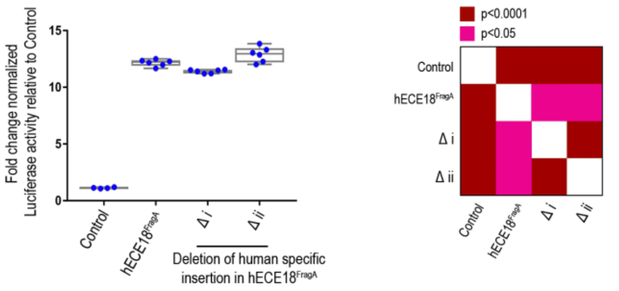

K Effect of mutagenizing derived human variants to ancestral ape on hECE18 activity

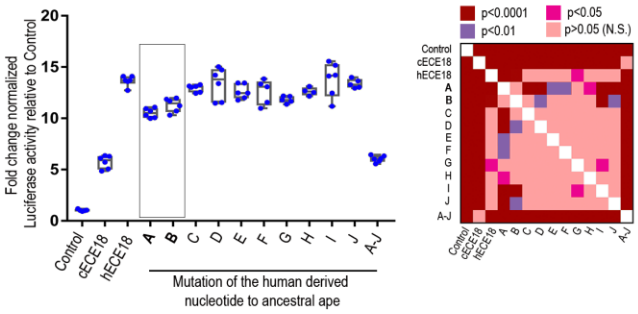

L Multiz alignment of placental mammals at SP1^A and SP1^B motifs

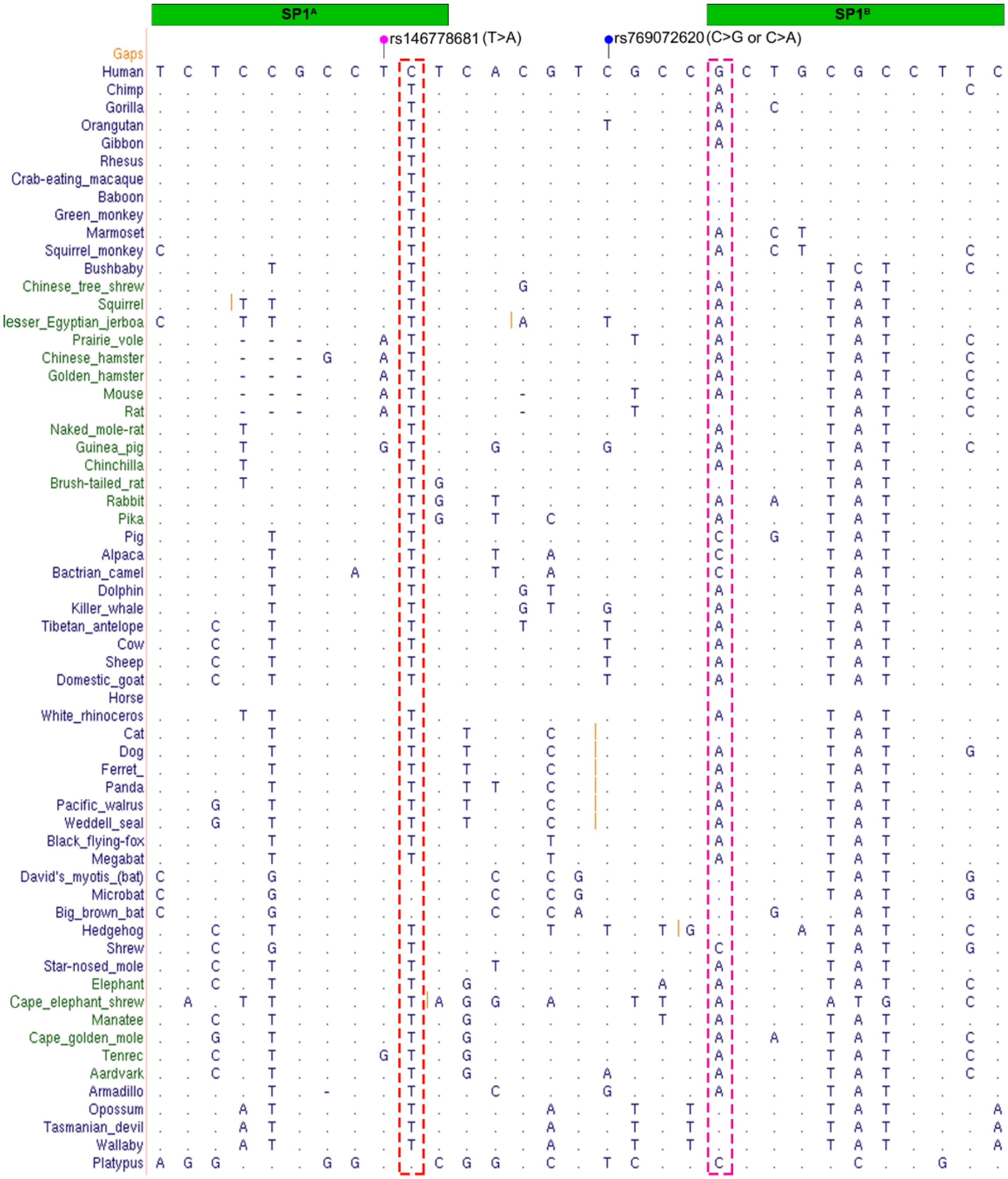

Fig. S3. Mapping ECE18 enhancer activity. (A) Multiz alignment of placental mammals centered on human ECE18 (hECE18). hECE18 was split into two fragments hECE18^FragA and hECE18^FragB. 2xHAR20 (dark grey) is a merged element that contains HACNS56, HAR19 and HAR80 (light grey) (6, 9, 10). Conserved elements used as kernels are (#124, #125, #126 and #127) shown in dark

blue. **(B)** Modified Stagia3 bidirectional luciferase lentiviral reporter vector used to test enhancer activity in cultured keratinocytes. LTR indicates long terminal repeat; ψ, packaging signal; RRE, rev response element; cPPT, central polypurine tract; SV40 polyA, simian vacuolating virus 40 polyadenylation signal; HSV TK promoter, Herpes simplex virus thymidine kinase promoter; cHS4core, insulator core derived from the chicken CHS4 element; Sequence to test; WPRE, woodchuck posttranscriptional regulatory element; bGH polyA, bovine growth hormone polyadenylation signal. **(C)** Coordinates of ECE18 mammalian homologs and fragments tested in this study. Genome builds are indicated. **(D)** Representative images of GFP antibody-stained sections from P2.5 forelimbs of hECE18^{FragA} lentivirus-mediated transgenic mouse. GFP positive clones are visualized by HRP-DAB coupled immunohistochemistry so positive clones appear brown (black arrows). Dorsal hair follicle (blue arrow). **(E)** Comparative quantitative activity of mammalian ECE18^{FragA} orthologs in cultured human GMA24F1A keratinocytes. Fold change normalized luciferase activity relative to Control (empty vector) is plotted. **(F)** Allele frequencies of polymorphic human variants rs56967129, rs146778681, rs769072620, rs529226880 are shown. Allele frequencies obtained from the 1000 Genomes Project Phase 3, gnomAD genomes r3.0 and UK10K datasets **(11–13)**. **(G)** Fold change normalized luciferase induction relative to Control (empty vector) by hECE18^{FragA} containing minor allele at rs56967129, rs146778681, rs769072620 and rs529226880. **(H)** Fold change luciferase quantitative activity of hECE18 human: chimp chimeric enhancers relative to Control (empty vector). Maps of the human-chimp chimeric enhancers are shown (box). **(I)** Genomic location and alignments of derived human single nucleotide substitutions (A-J), derived human-specific insertions (i and ii), and modern human polymorphic variants (rs56967129, rs146778681, rs769072620, rs529226880) within hECE18^{FragA} are shown. Coordinates in hg38. **(J)** Fold change normalized luciferase activity of hECE18^{FragA} after deletion of derived human insertions i and ii. **(K)** Fold change luciferase activity relative to Control (empty vector) upon mutagenesis of hECE18 human derived variants A-J alone or all together to ancestral ape base. **(L)** Multiz alignments of placental mammals centered on the SP1^{A} and SP1^{B} motifs. A or B human derived variants are highlighted in red and pink, respectively. Human variants rs146778681 and rs769072620 are indicated. In panels **(E, G, H, J, K)** each point represents an

individual biological replicate and the median (line), 25%-75% percentiles (box bounds) and min and max (whiskers) are plotted. Significance determined by one-way *ANOVA* and Tukey's-adjusted *P*-values are shown in heatmaps. All assays performed in cultured human GMA24F1A keratinocytes.

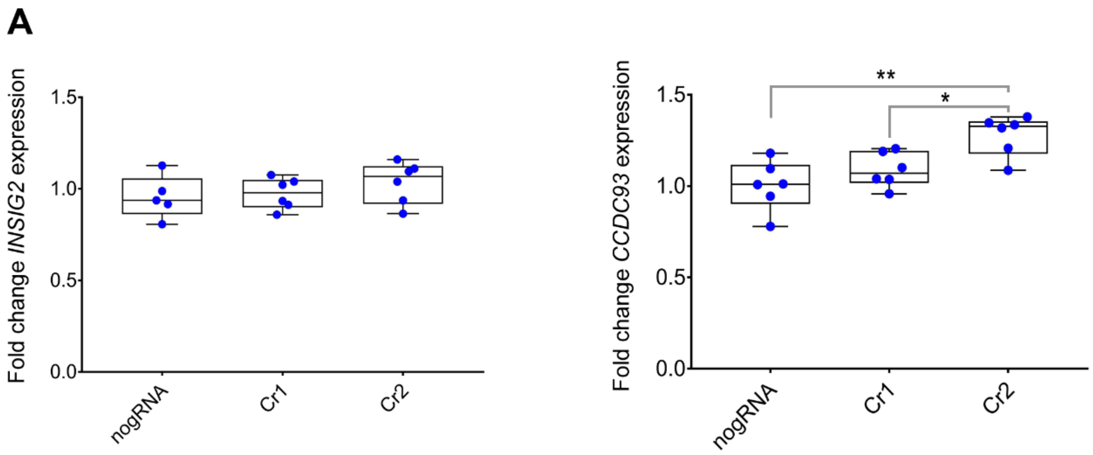

Fig S4. Effect of hECE18 repression on *INSIG2* and *CCDC93* expression in human keratinocytes. INSIG2 and CCDC93 are the only two protein-coding genes located within the EN1 topological associated domain (TAD). **(A)** Fold change expression of *INSIG2* and *CCDC93* upon dCas9-KRAB repression of hECE18 was assessed by qRT-PCR and calculated relative to dCAS9-KRAB transduction alone. Cr1 and Cr2 guide RNAs target hECE18. In graphs each point represents an individual biological replicate, median (line), 25%-75% percentiles (box bounds) and min and max (whiskers) are plotted and significance by one-way ANOVA. Tukey-adjusted P-values are reported. ** P<0.01, * P<0.05. Data normalized to the human β-ACTIN gene. Experiments performed in cultured human GMA24F1A keratinocytes.

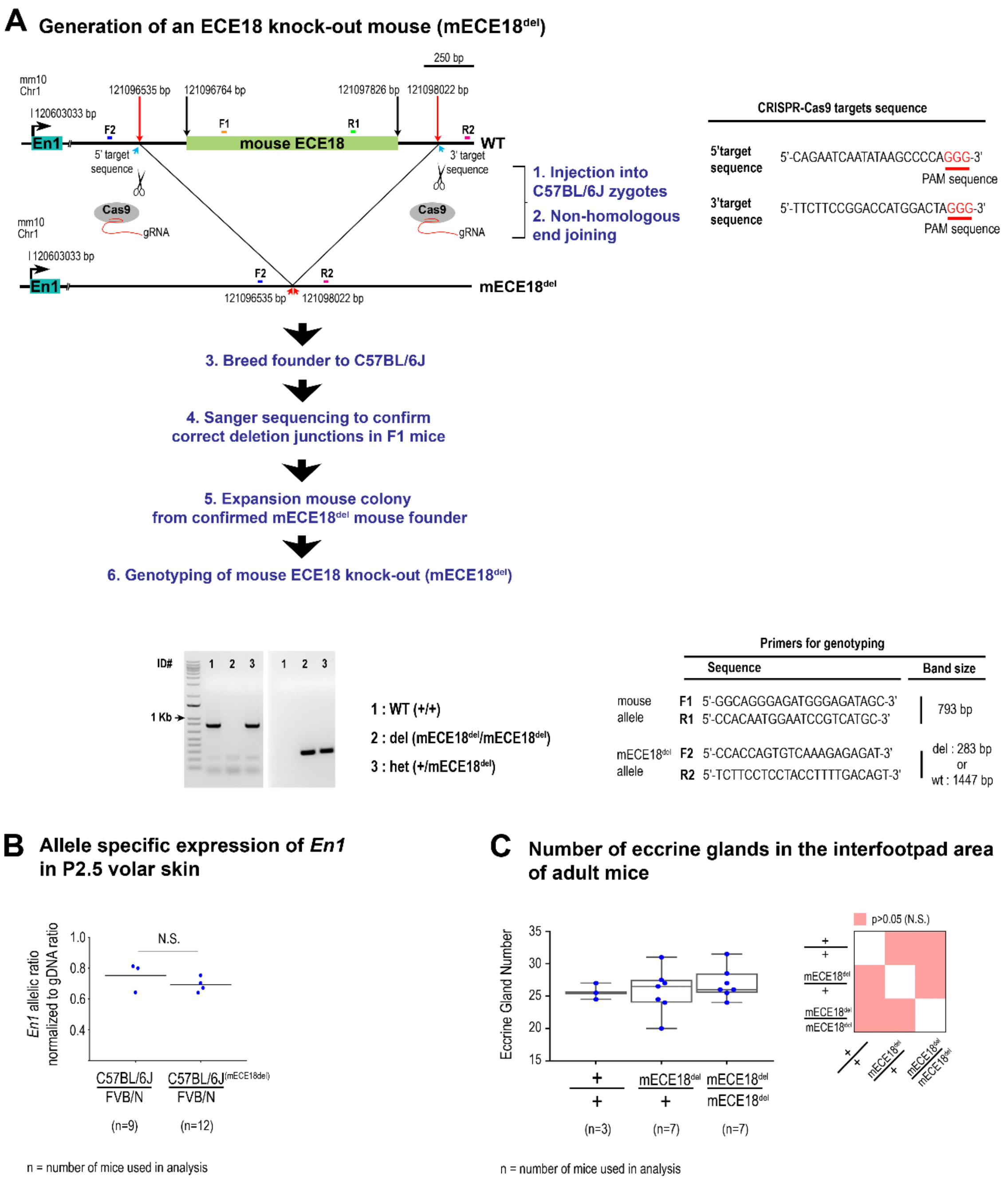

Fig. S5. Generation and characterization of volar phenotypes of ECE18 knock-out mouse. (A) Generation of an ECE18 knock-out mouse (mECE18^{del}) by CRISPR-Cas9 mediated genome editing. CRISPR-Cas9 target sequence and genotyping strategy are shown. Correct deletion junctions were confirmed by Sanger sequencing of F1 pups. **(B)** Normalized ratio of C57BL/6J : FVB/N allelic expression of *En1* from P2.5 volar forelimb of wildtype (C57BL/6J : FVB/N) and

mECE18del (C57BL/6J$^{(mECE18del)}$: FVB/N) F1 hybrid mice. Ratios are normalized to the allelic ratio in F1 genomic DNA. Each point represents the mean value across three technical replicates for three or four biological samples consisting of pooled P2.5 volar skins from both forelimbs of three mice. **(C)** Quantification of the number of eccrine glands in the forelimb IFP of adult wildtype (+/+), mECE18^{del} heterozygous (+/mECE18^{del}) and homozygous mECE18^{del}/ mECE18^{del} mice. Each point represents the average number of IFP eccrine glands across both forelimbs of an individual mouse. The total number of animals analyzed per genotype (n). In panel **(B)** significance was assessed by a student's T-test (two-tailed). In panel **(C)** significance assessed by one-way *ANOVA*. Tukey-adjusted *P*-values are shown in a heatmap and the median (line), 25%-75% percentiles (box bounds) and min and max (whiskers) are plotted. N.S., not significant.

A Generation of a human ECE18 knock-in (hECE18^{KI}) mouse

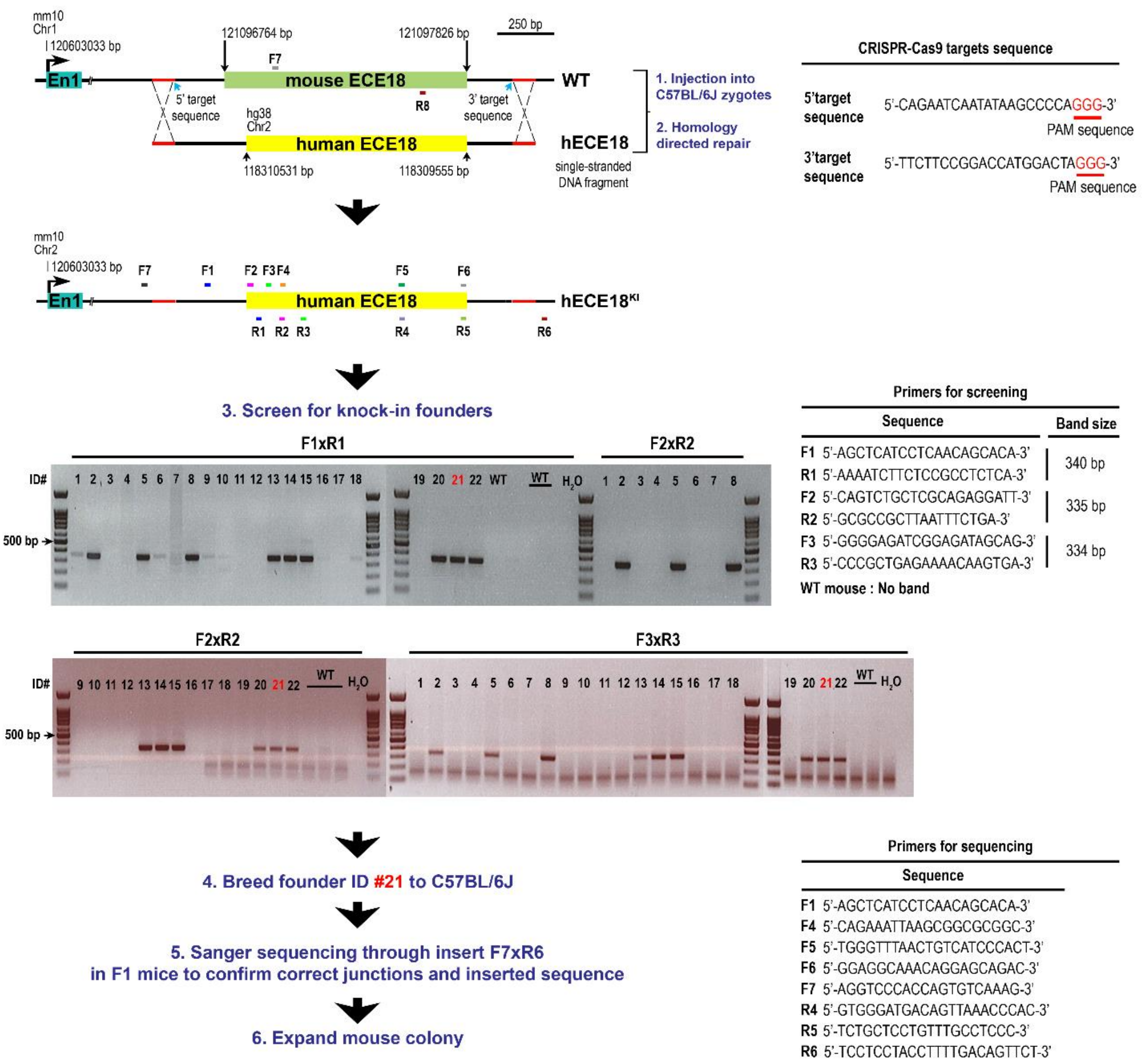

B Genotyping of human ECE18 knock-in (hECE18^{KI}) mouse

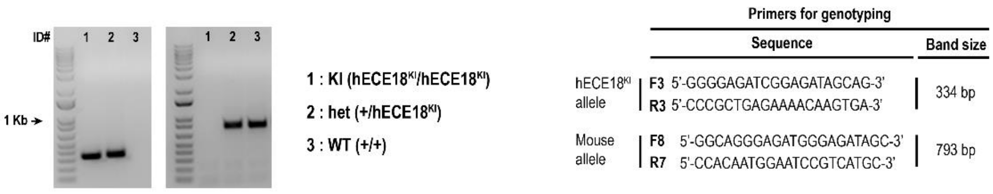

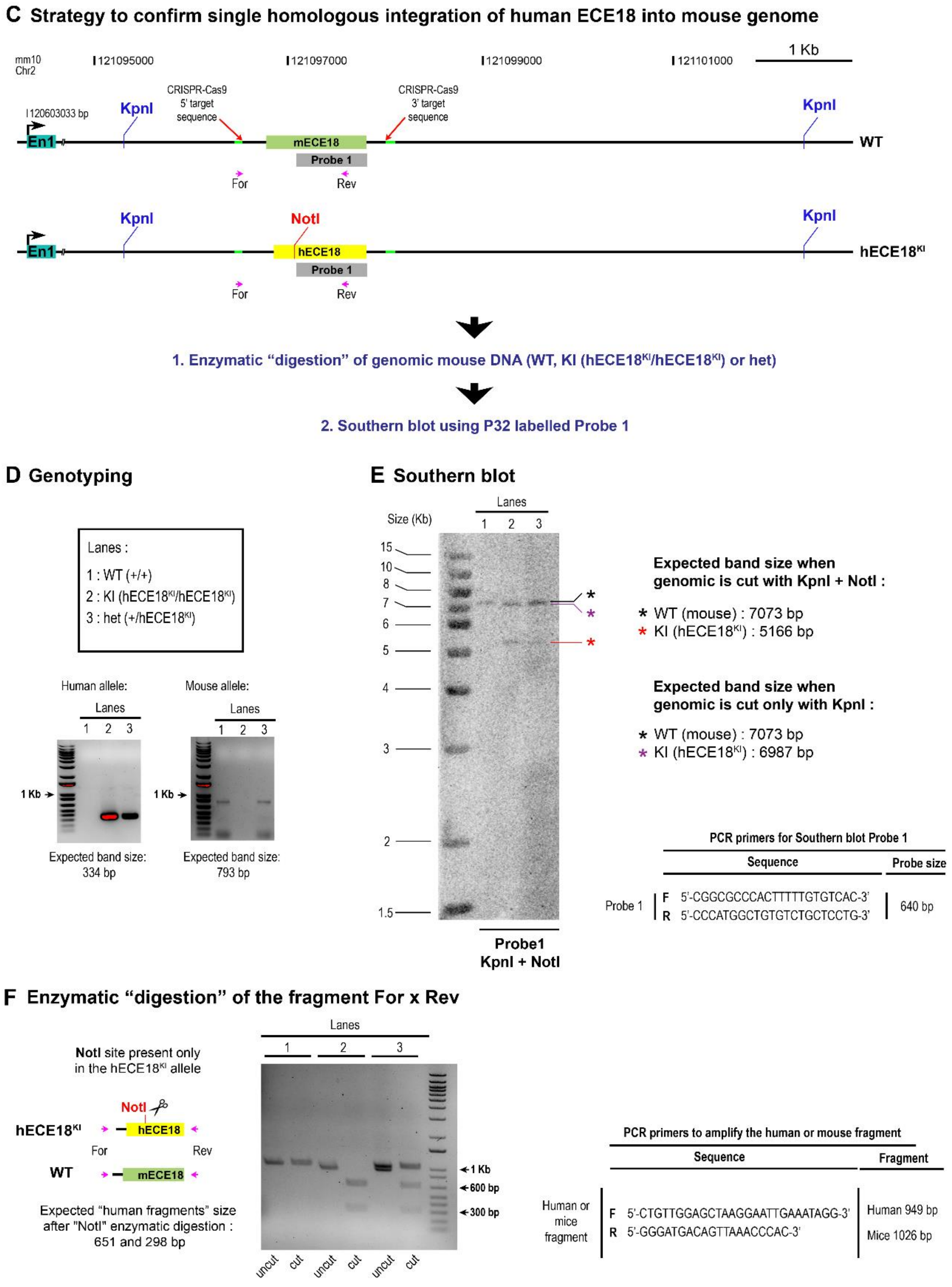

Fig. S6. Generation of human ECE18 knock-in (hECE18KI) mice. (A) Detailed overview of the generation of a human ECE18 knock-in mouse (hECE18KI). CRISPR-Cas9 technology was used

to replace the endogenous mouse ECE18 with the orthologous human ECE18 sequence. A single knock-in founder mouse (ID #21) was identified and bred to a C57BL/6J male generate F1 pups. F1 pups were screened to confirm transmission of the knock-in allele and correct targeting in 2 pups validated to have correct junctions and insert sequence were used to generate two $hECE18^{KI}$ lines. Each founder F1 mouse was bred onto C57BL/6J for two more generations prior to phenotypic analyses. Phenotypic analyses reported are based on progeny derived from both F1 founder lines at the N3 generation. CRISPR-Cas9 targets sequence, and primers used are listed. **(B)** Representative agarose gel for genotyping $hECE18^{KI}$ mice. Sequences of genotyping primers used are shown. **(C)** Schematic of secondary Southern and long-range PCR/species specific restriction digest strategies to validate single homologous integration of hECE18 into $hECE18^{KI}$ mice. KpnI and NotI sites are shown. **(D)** Genotyping PCR to determine genotype of mice used as source of genomic DNA for the southern blot in E and long-range PCR in F. Primers for genotyping listed in B and are species specific. **(E)** Autoradiograph of genomic southern blot using P32 labelled Probe 1 is shown. Sequences of primers used to amplify Probe 1 are shown and Probe target sequence is shown in C. Due to sequence conservation, probe against ECE18 (Probe1) cannot distinguish between mouse and human sequence, however hECE18 contains a unique NotI site that is not present in the orthologous mouse ECE18 genomic sequence. Thus, digest with NotI was used to validate integration of hECE18 in KI and het mouse genomic DNA. Presence of uncut KpnI fragment in KI and het lanes is due to incomplete NotI digest of genomic DNA. This is likely the result of partial CpG methylation of the hECE18 sequence, which blocks NotI cleavage, and is consistent with the observation that the enhancer is inactive in some tissues used as the source material for genomic DNA (tail biopsies). **(F)** A secondary strategy was used in F to confirm identify of sequence within swapped region. Enzymatic digestion of the long-range PCR amplified DNA fragment from WT, KI ($hECE18^{KI}$/ $hECE18^{KI}$) and het (+/$hECE18^{KI}$) mice. Primers used to amplify the genomic DNA fragments are listed. NotI enzyme cut uniquely in the human fragment. WT, wild type. KI, hECE18 knock-in homozygote. het, hECE18 KI heterozygote.

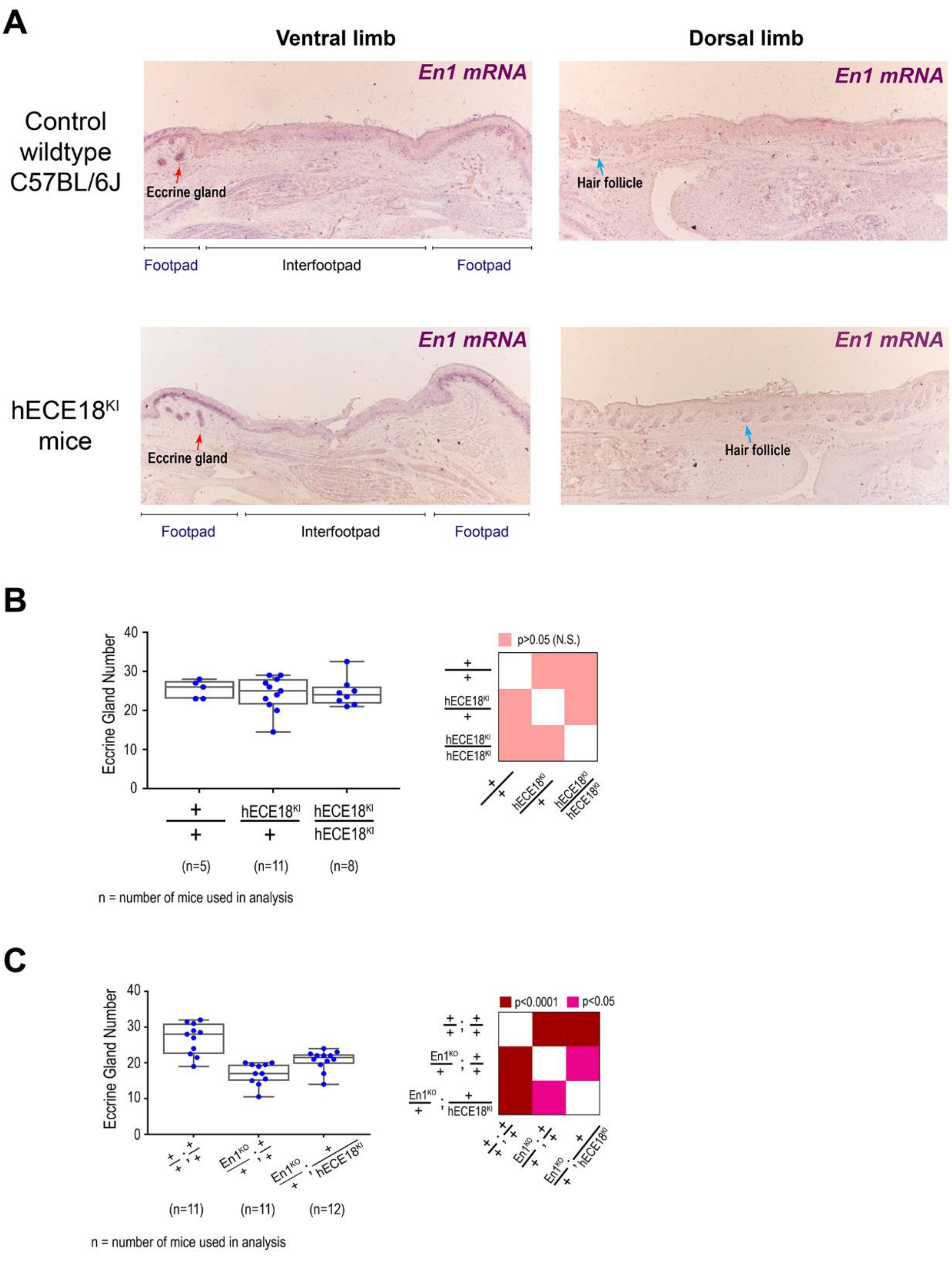

Fig. S7. Effect of hECE18^{KI} and En1^{KO} alleles on mouse *En1* expression and interfootpad eccrine gland number. (A) Spatial *En1* mRNA expression in Control (+/+) and hECE18^{KI} (hECE18^{KI}/hECE18^{KI}) mice. Representative images of the ventral limb, and the hairy dorsal limb at P2.5 stage is shown for both genotypes. *En1* mRNA transcripts are detected using anti-DIG alkaline

phosphatase coupled antibody and appear in purple. **(B)** Effect of hECE18 on IFP eccrine gland number in a wildtype genetic background. IFP eccrine gland number in the forelimb adult wildtype (+/+), +/hECE18^{KI} and hECE18^{KI}/hECE18^{KI} mice is plotted. **(C)** Effect of En1^{KO} allele on eccrine gland number. The number of eccrine glands in the forelimb IFP of adult +/+; +/+, En1^{KO}/+; +/+, and En1^{KO}/+; +/hECE18^{KI} mice is plotted. Values for animals carrying En1^{KO} are also reported in the main text in Fig.3g. In panels **(B, C)** each point represents the average number of IFP eccrine glands across both forelimbs of an individual mouse and median (line), 25%-75% percentiles (box bounds) and min and max (whiskers) are plotted. The total number of animals analyzed per genotype (n). Significance was assessed by one-way *ANOVA* and Tukey-adjusted *P*-values are reported in heatmaps. +, wildtype allele. KI, knock-in. KO, knock-out. N.S., not significant.

Table S1: Primers used to subclone ECEs in mouse transgenic assays#

ECE	Forward sequence	Reverse sequence
mouse-ECE1	accaattgctcgaggGTGCATTTATCATCCTATTACT	gtcaagcttccattatatagTTACACAGAGGCTAGATTGG
mouse-ECE2	tcgatagtcgaccaattgctcgaggCCCAAATCCCTGCCGCTTCT	cccttgctcaccatggtggcTCTTTGCTTTTTTGCAGGGAGAGCGC
mouse-ECE3	accaattgctcgaggCCTTTGCTGGAGCACCTA	gtcaagcttccattatatagGTCACTCCTCCACATTAA
mouse-ECE4	accaattgctcgaggCAATCAGGGCGACAG	gtcaagcttccattatatagTGGTCGCCACACAGTA
mouse-ECE5	accaattgctcgaggGAGAACTGCCTGTCCCGT	gtcaagcttccattatatagCACCAGGCTCTGTATGTA
mouse-ECE6	cgaccaattgctcgaggCTAGGAGACAGCCTTCCTGGAGG	cggtcaagcttccattatatagTGGCATGCCCTGCAAGTCTGAC
mouse-ECE7	accaattgctcgaggGTACTCACTGGATCTGAA	gtcaagcttccattatatagACTGCCTGAGTCATCTCC
mouse-ECE8	accaattgctcgaggATAACTGAGTTTGGTTTA	gtcaagcttccattatatagTGCTCAGCCCCTCCTCAG
mouse-ECE9	accaattgctcgaggACTGAGCTACATCCGTGG	gtcaagcttccattatatagTCTGTCATATACTTCTCG
mouse-ECE10	accaattgctcgaggCAGAACCTTCTGTATTTA	gtcaagcttccattatatagCAAGTGCTCTCTCACAGG
mouse-ECE11	agtcgaccaattgctcgaggCTCAGTGAGTCTCTGACAAGCC	tccggtcaagcttccattatatagGTGATTCTCTGACCTCTGCATGC
mouse-ECE12	accaattgctcgaggGCTCCTCTTTCCCAACAA	gtcaagcttccattatatagCATGATTGTCACTGGTCC
mouse-ECE13	accaattgctcgaggCTTATTGATTGGATATATG	gtcaagcttccattatatagCCAGTGAGCATAGCAGTGG
mouse-ECE14	agtcgaccaattgctcgaggTGAGGACCTGAGTTCAAATCCC	tccggtcaagcttccattatatagCCATATAGACAGGCACATGCAC
mouse-ECE15	accaattgctcgaggCCTTTGCAGACCTAGAAT	gtcaagcttccattatatagGGAGAACAAACCACCCAG
mouse-ECE16	agtcgaccaattgctcgaggCCATCCTGAGTCAAGGGCGG	gtcaagcttccattatatagCTTCAGAACTCAGAGTAGGGTCAAGC
mouse-ECE17	accaattgctcgaggCATGCATTATATTTACATCA	gtcaagcttccattatatagGGAAGTACCAATGAATCACA
mouse-ECE18	agtcgaccaattgctcgaggTGTGTTTGCCTCCTACAATGGGAGGATA	gtcaagcttccattatataGCTGAAGTTTTCCTTTCTGTTTACCAGGAAG
chimp-ECE18	accaattgctcgaggCCTGTTTGCCTCCCATAATA	gtcaagcttccattatatagCAGAAGTTTTCTTTTCTGTT
human-ECE18	accaattgctcgaggCCTGTTTGCCTCCCATAATA	gtcaagcttccattatatagCAGAAGTTTTCTTTTCTGTT
mouse-ECE19	agtcgaccaattgctcgaggTGTTGGAATAAGGGCACACC	tccggtcaagcttccattatatagGACACTGCTGCCTTCTCTATTC
mouse-ECE20	accaattgctcgaggCACATTCAAGGTCAATG	gtcaagcttccattatatagGCAGCAGTGAGTGTG
mouse-ECE21	accaattgctcgaggCACAATTGCCTCTTTTAGGT	gtcaagcttccattatatagCAAGGATGTTTTCAAATTAG
mouse-ECE22	agtcgaccaattgctcgaggCCTGACCTATCTGCCATCTCC	tccggtcaagcttccattatatagGCCTACACGTTAAGCAATAGGGT
mouse-ECE23	accaattgctcgaggTCTCTGCATAACAGCCCT	gtcaagcttccattatatagGCACTTTAATCACTAAGC

#Lower case sequence indicates homology arms to Stagia3 vector

Table S2: Primers used to clone ECE18 orthologs into bidirectional luciferase reporter vector#

ECE	Forward sequence	Reverse sequence
cat-ECE18	agagatttagaatgacaggcGAGGGATAGGAATAGAAAAGGCCC	tcaagcttccattatatagaattccAAGTTTTCTTTGCTGTTTACCGG
cat-ECE18-FragA	gaaagagagatttagaatgacaggcCAACATATCGGGCTTACAAATTATC	tcaagcttccattatatagaattccAAGTTTTCTTTGCTGTTTACCGG
mouse-ECE18	agagatttagaatgacaggcTGTGTTTGCCTCCTACAATGGG	gcttccattatatagaattccCTGAAGTTTTCCTTTCTGTTTACCAGG
mouse-ECE18-FragA	gaaagagagatttagaatgacaggcCAACATATCGCGCTTACAAATTATC	gcttccattatatagaattccCTGAAGTTTTCCTTTCTGTTTACCAGG
bushbaby-ECE18	agagatttagaatgacaggcCGTGTTTGCCTCCCACAATAAGAGG	cttccattatatagaattccGGAAAGTTTTCTTTTCAGTTTACCAAGGAG
marmoset-ECE18	agagatttagaatgacaggcCCTGTTTGCCTCCCACAATAAGAG	cttccattatatagaattccCAAAAGTTTTCTTTTCTGTTTACCGGGG
macaque-ECE18	agagatttagaatgacaggcCCTGTTTGCCTCCCATAGTAAGAGG	agcttccattatatagaattccCAAAAGTTTTCTTTTCTGTTTGTCGGGG
macaque-ECE18-FragA	agagatttagaatgacaggcCAACATATCGCGCTTACAAATTAT	agcttccattatatagaattccCAAAAGTTTTCTTTTCTGTTTGTCGGGG
gorilla-ECE18-FragA	agagatttagaatgacaggcCAACATATCGCGCTTACAAATTAT	cttccattatatagaattccCAGAAGTTTTCTTTTCTGTTTACCGGG
chimp-ECE18	agagatttagaatgacaggcCCTGTTTGCCTCCCATAATAAGAGG	cttccattatatagaattccCAGAAGTTTTCTTTTCTGTTTACCGGG
chimp-ECE18-FragA	agagatttagaatgacaggcCAACATATCGCGCTCGCAAA	cttccattatatagaattccCAGAAGTTTTCTTTTCTGTTTACCGGG
human-ECE18	agagatttagaatgacaggcCCTGTTTGCCTCCCATAATAAGAGG	cttccattatatagaattccCAGAAGTTTTCTTTTCTGTTTACCGGG
human-ECE18-fragA	agagatttagaatgacaggcCAACATATCGCGCTTACAAATTAT	agcttccattatatagaattccCAGAAGTTTTCTTTTCTGTTTACCGGGGAGGGGAG
human-ECE18-fragB	agagatttagaatgacaggcCCTGTTTGCCTCCCATAATAAGAGG	gtcaagcttccattatatagaattccTTGTTTTCTCAGCGGGCCCG

#Lowercase sequence indicates homology arm to vector.

Table S3: Primers used mutagenesis of ECE18#

ECE	Unique primer
hECE18-Mut-A	GGAAATGAAAATCTTCTCCGCCTtTCACGTCGCCGCTGCG
hECE18-Mut-B	CGCCTCTCACGTCGCCaCTGCGCCTTCAAATCCTCTGC
hECE18-Mut-C	CCTCACCTAATGCAAATGGAaCGGAGGCCTCTGTTATTGTATT
hECE18-Mut-D	CCTCACCTAATGCAAATGGAGtGGAGGCCTCTGTTATTGTATT
hECE18-Mut-E	TTAATCCGGCTTAGCtCGCTTAATGATGCCA
hECE18-Mut-F	AATCCGGCTTAGCCCaCTTAATGATGCCACT
hECE18-Mut-G	ATCGGGCCCGCTGAGAAAAtAAGTGACACAAAAAGTGGGCG
hECE18-Mut-H	CGCCCGCGATGGCGCtGATGGCTGATGCCGCGATTACGCC
hECE18-Mut-I	CTATCTCCGATCTCCCCGCCaGGTTTTTCATACTGATATTCTTTGCACCCC
hECE18-Mut-J	GGTTTTTCATACTGATATTaTTTGCACCCCGTAACACAGG
hECE18-FragA-rs56967129 C>T	TTTCATACTGATATTtTTTGCACCCCGTAAC
hECE18-FragA-rs146778681 T>A	AAAATCTTCTCCGCCaCTCACGTCGCCGCTG
hECE18-FragA-rs769072620 C>G	CTCCGCCTCTCACGTgGCCGCTGCGCCTTC
hECE18-FragA-rs769072620 C>A	CTCCGCCTCTCACGTaGCCGCTGCGCCTTCA
hECE18-FragA-rs529226880 C>T	CCTTCAAATCCTCTGtGAGCAGACTGGCCTC
hECE18-del-insertion_i	CGGCCCCCTCCCGGGCTCCCTCCCCGGTAAACAGAAAAGA
hECE18-del-insertion_ii	GAAGAGATTTATATTTTTTTGTTGTCAGGAAATGAACAAA
gECE18-Mut1	GCGGAGAAGTCTATgAACTACTTCCATTAAAATGC
gECE18-Mut2	CGCCGCCCGCGATGGCGCtGgTGGCTGATGCCGCG
gECE18-Mut3	TGAAGAGATTTATATTTTTTTGTTGTCAGGAAATG
gECE18-Mut4	CTTCTCCGCCTtTCACGTCGCCaCcGCGCCTTCAA
gECE18-Mut5	CACCGGCCCCtTCCCGGGCTCCCTCCCCGGTAAAC
hECE18_FragA-del_in_human_SP1A	CCCGTAACACAGGAAATGAAAATCTCACGTCGCCGCTGCGCCTTC
hECE18_FragA-del_in_human_SP1B	CTTCTCCGCCTCTCACGTCGCCAAATCCTCTGCGAGCAGACTGG
mECE18_FragA-add_in_mouse_hSP1A_B	GAAATGAAAATCTTCTCCGCCTCTCACGTCGCCGCTGCGCCTTCAAATCCTCTGCGAACA
mECE18_FragA-add_in_mouse_hSP1A	TGAAAATCTTCTCCGCCTCTCAGTCTCCACTGTATCTCCAAATCC
mECE18_FragA-add_in_mouse_hSP1B	CAGGAAATGAAAATCTTCTCCATTCACGTCGCCGCTGCGCCTTCAAATCCTCTGCGAACA
Chimera-5’-human-chimp-3’-F	CGCCGATGGCTGATGCCGCGATTACGCCGGCGGGGGCGGCCCGC
Chimera-5’-human-chimp-3’-R	GCCGCCCCCGCCGGCGTAATCGCGGCATCAGCCATCGGCGCCATC
Chimera-5’-chimp-human-3’-F	CGCTGATGGCTGATGCCGCGATTACGCCGGCGGGGGCGGCCCGC
Chimera-5’-chimp-human-3’-F	GCCGCCCCCGCCGGCGTAATCGCGGCATCAGCCATCAGCGCCAT

#Lowercase nucleotide indicates the base changed in mutagenesis.

Table S4: ChIP-qPCR and qRT-PCR primer sequences

Name	Specie	Forward sequence	Reverse sequence
SP1A/B (hECE18)	Human	TGAAAATCTTCTCCGCCTCTCACG	CCAGTCTGCTCGCAGAGGAT
HBG2 promoter	Human	CCAAGGTCATGGATCGAGTT	ACACTGTGACAGCTGGGATG
En1	Mouse	GTGGTCAAGACTGACTCACAGC	GCTTGTCTTCCTTCTCGTTCTT
Rpl13a	Mouse	CAGTGCGCCAGAAAATGC	GAAGGCATCAACATTTCTGGAA
EN1	Human	TTCGGATCGTCCATCCTCC	GCTCCGTGATGTAGCGGTTT
INSIG2	Human	TTGCTGGAGGCATAACAATGGG	TGCCTTCTTCATTCCTGATGAGATT
CCDC93	Human	TGAACGACCAGTACTTGGAGCTG	GGATGTTCAGGAGGCCTTCG
Beta-ACTIN	Human	CATGTACGTTGCTATCCAGGC	CTCCTTAATGTCACGCACGAT

SI References

1. G. Andrey, *et al.*, Characterization of hundreds of regulatory landscapes in developing limbs reveals two regimes of chromatin folding. *Genome Res.* **27**, 223–233 (2017).

2. C. A. Davis, *et al.*, The Encyclopedia of DNA elements (ENCODE): data portal update. *Nucleic Acids Res.* **46**, D794–D801 (2018).

3. D. Aldea, *et al.*, The Transcription Factor Deaf1 Modulates Engrailed-1 Expression to Regulate Skin Appendage Fate. *J. Invest. Dermatol.* **139**, 2378-2381.e4 (2019).

4. J. A. Capra, G. D. Erwin, G. McKinsey, J. L. R. Rubenstein, K. S. Pollard, Many human accelerated regions are developmental enhancers. *Philos. Trans. R. Soc. Lond., B, Biol. Sci.* **368**, 20130025 (2013).

5. K. S. Pollard, *et al.*, Forces shaping the fastest evolving regions in the human genome. *PLoS Genet.* **2**, e168 (2006).

6. S. Prabhakar, J. P. Noonan, S. Pääbo, E. M. Rubin, Accelerated evolution of conserved noncoding sequences in humans. *Science* **314**, 786 (2006).

7. S. Joost, *et al.*, Single-Cell Transcriptomics Reveals that Differentiation and Spatial Signatures Shape Epidermal and Hair Follicle Heterogeneity. *cels* **3**, 221-237.e9 (2016).

8. S. Liu, H. Zhang, E. Duan, Epidermal Development in Mammals: Key Regulators, Signals from Beneath, and Stem Cells. *International Journal of Molecular Sciences* **14**, 10869–10895 (2013).

9. J. A. Capra, G. D. Erwin, G. McKinsey, J. L. R. Rubenstein, K. S. Pollard, Many human accelerated regions are developmental enhancers. *Philos Trans R Soc Lond B Biol Sci* **368** (2013).

10. K. S. Pollard, *et al.*, Forces Shaping the Fastest Evolving Regions in the Human Genome. *PLoS Genet* **2** (2006).

11. 1000 Genomes Project Consortium, *et al.*, A global reference for human genetic variation. *Nature* **526**, 68–74 (2015).

12. K. J. Karczewski, *et al.*, The mutational constraint spectrum quantified from variation in 141,456 humans. *Nature* **581**, 434–443 (2020).

13. UK10K Consortium, *et al.*, The UK10K project identifies rare variants in health and disease. *Nature* **526**, 82–90 (2015).
